# Chemolithoautotrophic diazotrophs dominate dark nitrogen fixation in mangrove sediments

**DOI:** 10.1093/ismejo/wrae119

**Published:** 2024-06-25

**Authors:** Shasha Wang, Lijing Jiang, Zhuoming Zhao, Zhen Chen, Jun Wang, Karine Alain, Liang Cui, Yangsheng Zhong, Yongyi Peng, Qiliang Lai, Xiyang Dong, Zongze Shao

**Affiliations:** Key Laboratory of Marine Genetic Resources, Third Institute of Oceanography, Ministry of Natural Resources of China; State Key Laboratory Breeding Base of Marine Genetic Resources; Fujian Keey Laboratory of Marine Genetic Resources; Sino-French Laboratory of Deep-Sea Microbiology (MicrobSea), Xiamen 361005, PR China; Key Laboratory of Marine Genetic Resources, Third Institute of Oceanography, Ministry of Natural Resources of China; State Key Laboratory Breeding Base of Marine Genetic Resources; Fujian Keey Laboratory of Marine Genetic Resources; Sino-French Laboratory of Deep-Sea Microbiology (MicrobSea), Xiamen 361005, PR China; Key Laboratory of Marine Genetic Resources, Third Institute of Oceanography, Ministry of Natural Resources of China; State Key Laboratory Breeding Base of Marine Genetic Resources; Fujian Keey Laboratory of Marine Genetic Resources; Sino-French Laboratory of Deep-Sea Microbiology (MicrobSea), Xiamen 361005, PR China; Key Laboratory of Marine Genetic Resources, Third Institute of Oceanography, Ministry of Natural Resources of China; State Key Laboratory Breeding Base of Marine Genetic Resources; Fujian Keey Laboratory of Marine Genetic Resources; Sino-French Laboratory of Deep-Sea Microbiology (MicrobSea), Xiamen 361005, PR China; Key Laboratory of Marine Genetic Resources, Third Institute of Oceanography, Ministry of Natural Resources of China; State Key Laboratory Breeding Base of Marine Genetic Resources; Fujian Keey Laboratory of Marine Genetic Resources; Sino-French Laboratory of Deep-Sea Microbiology (MicrobSea), Xiamen 361005, PR China; Univ Brest, CNRS, Ifremer, EMR6002 BIOMEX, Biologie Interactions et adaptations des Organismes en Milieu EXtrême, IRP 1211 MicrobSea, F-29280 Plouzané, France; Key Laboratory of Marine Genetic Resources, Third Institute of Oceanography, Ministry of Natural Resources of China; State Key Laboratory Breeding Base of Marine Genetic Resources; Fujian Keey Laboratory of Marine Genetic Resources; Sino-French Laboratory of Deep-Sea Microbiology (MicrobSea), Xiamen 361005, PR China; Key Laboratory of Marine Genetic Resources, Third Institute of Oceanography, Ministry of Natural Resources of China; State Key Laboratory Breeding Base of Marine Genetic Resources; Fujian Keey Laboratory of Marine Genetic Resources; Sino-French Laboratory of Deep-Sea Microbiology (MicrobSea), Xiamen 361005, PR China; Key Laboratory of Marine Genetic Resources, Third Institute of Oceanography, Ministry of Natural Resources of China; State Key Laboratory Breeding Base of Marine Genetic Resources; Fujian Keey Laboratory of Marine Genetic Resources; Sino-French Laboratory of Deep-Sea Microbiology (MicrobSea), Xiamen 361005, PR China; Key Laboratory of Marine Genetic Resources, Third Institute of Oceanography, Ministry of Natural Resources of China; State Key Laboratory Breeding Base of Marine Genetic Resources; Fujian Keey Laboratory of Marine Genetic Resources; Sino-French Laboratory of Deep-Sea Microbiology (MicrobSea), Xiamen 361005, PR China; Key Laboratory of Marine Genetic Resources, Third Institute of Oceanography, Ministry of Natural Resources of China; State Key Laboratory Breeding Base of Marine Genetic Resources; Fujian Keey Laboratory of Marine Genetic Resources; Sino-French Laboratory of Deep-Sea Microbiology (MicrobSea), Xiamen 361005, PR China; Southern Marine Science and Engineering Guangdong Laboratory (Zhuhai), Zhuhai 519000, PR China; Key Laboratory of Marine Genetic Resources, Third Institute of Oceanography, Ministry of Natural Resources of China; State Key Laboratory Breeding Base of Marine Genetic Resources; Fujian Keey Laboratory of Marine Genetic Resources; Sino-French Laboratory of Deep-Sea Microbiology (MicrobSea), Xiamen 361005, PR China; Southern Marine Science and Engineering Guangdong Laboratory (Zhuhai), Zhuhai 519000, PR China

**Keywords:** nitrogen fixation, chemolithoautotroph, Campylobacterota, mangrove, sediment, metagenomic, metatranscriptomic

## Abstract

Diazotrophic microorganisms regulate marine productivity by alleviating nitrogen limitation. So far chemolithoautotrophic bacteria are widely recognized as the principal diazotrophs in oligotrophic marine and terrestrial ecosystems. However, the contribution of chemolithoautotrophs to nitrogen fixation in organic-rich habitats remains unclear. Here, we utilized metagenomic and metatranscriptomic approaches integrated with cultivation assays to investigate the diversity, distribution, and activity of diazotrophs residing in Zhangzhou mangrove sediments. Physicochemical assays show that the studied mangrove sediments are typical carbon-rich, sulfur-rich, nitrogen-limited, and low-redox marine ecosystems. These sediments host a wide phylogenetic variety of nitrogenase genes, including groups I–III and VII–VIII. Unexpectedly diverse chemolithoautotrophic taxa including *Campylobacteria*, *Gammaproteobacteria*, *Zetaproteobacteria*, and *Thermodesulfovibrionia* are the predominant and active nitrogen fixers in the 0–18 cm sediment layer. In contrast, the 18–20 cm layer is dominated by active diazotrophs from the chemolithoautotrophic taxa *Desulfobacterota* and *Halobacteriota*. Further analysis of MAGs shows that the main chemolithoautotrophs can fix nitrogen by coupling the oxidation of hydrogen, reduced sulfur, and iron, with the reduction of oxygen, nitrate, and sulfur. Culture experiments further demonstrate that members of chemolithoautotrophic *Campylobacteria* have the nitrogen-fixing capacity driven by hydrogen and sulfur oxidation. Activity measurements confirm that the diazotrophs inhabiting mangrove sediments preferentially drain energy from diverse reduced inorganic compounds other than from organics. Overall, our results suggest that chemolithoautotrophs rather than heterotrophs are dominant nitrogen fixers in mangrove sediments. This study underscores the significance of chemolithoautotrophs in carbon-dominant ecosystems.

## Introduction

Nitrogen serves as an essential component of all living organisms, constituting the main nutrient limiting life on our planet [1, 2]. Nitrogen-fixing (diazotrophic) bacteria and archaea convert atmospheric dinitrogen gas (N_2_) into ammonia (NH_3_) for assimilation, which is mediated by three types of nitrogenases, including molybdenum–iron nitrogenase Nif (Mo–Fe), vanadium–iron nitrogenase Vnf (V–Fe), and iron-only nitrogenase Anf (Fe–Fe) [3, 4]. Biological nitrogen fixation counteracts the removal of bioavailable N by microbial denitrification and anaerobic ammonium oxidation, and provides a source of N to the majority of the biosphere that cannot directly assimilate N_2_ [5–7]. In particular, marine diazotrophs supply nearly one-half of the global fixed nitrogen demand, and their activity often regulates marine primary productivity [8, 9]. However, despite the well-documented ecological and biogeochemical importance of nitrogen fixation in the oceans [2, 10, 11], due to the high energy demand, only a few bacterial and archaeal populations have been shown to potentially fix nitrogen [12].

In oligotrophic marine environments, chemolithoautotrophs are considered important nitrogen fixers and can enhance the productivity of localized habitats [13]. For example, chemolithoautotrophic sulfur-oxidizing diazotrophs are identified as the key carbon and nitrogen providers to their symbiotic hosts, such as lucinid clams and cold water corals [14–16]. The ability to fix nitrogen has also been demonstrated in deep-sea anaerobic methane-oxidizing archaea from cold seep sediments [17] and methanogenic archaea from hydrothermal vent fluids [18]. In addition, chemolithoautotrophic nitrogen fixation has been well studied in oligotrophic terrestrial environments [13, 19]. *Cyanobacteriota* are considered the most important diazotrophs in glaciated forefields [20]. In mine tailings, it is postulated that chemolithoautotrophic diazotrophs utilize reduced sulfur compounds as electron donors for energy production [19]. Additionally, *Beggiatoa*-related chemolithoautrotrophic sulfur-oxidizing bacteria are suggested to actively fix nitrogen in oligotrophic sulfidic caves [14, 21]. In contrast, in organic-rich marine ecosystems, nitrogen fixation is often associated with heterotrophs [22–24]. For instance, the process of nitrogen fixation coupled with heterotrophic sulfate reduction has been documented in the sediments of Eckernförde Bay (Baltic Sea) and Narragansett Bay (Rhode Island) [25, 26]. Several heterotrophic taxa within the phyla *Pseudomonadota*, *Chloroflexota*, *Bacteroidota*, *Desulfobacterota*, and *Spirochaetota* are reported as the main diazotrophs in sedimentary ecosystems [27, 28]. These previous studies indicate that chemolithoautotrophs are the main nitrogen fixers in oligotrophic environments, whereas biological nitrogen fixation in organic-rich environments is mainly achieved by heterotrophs.

Mangrove sediments are typically considered organic-rich but nitrogen-limited ecosystems [29, 30]. Early research revealed a high rate of biological nitrogen-fixing activity mediated by microbes in the surface sediments of mangroves [31–33]. Recent work further indicated that nitrogen fixation rates increased with sediment depth from the surface to 100 cm in mangrove ecosystems [34]. Some heterotrophic prokaryotes in the phyla *Pseudomonadota* (classes *Alpha*- and *Gamma*-*proteobacteria*), *Desulfobacterota*, *Myxococcota*, and *Bacteroidota* were detected as the prevalent diazotrophs in mangrove sediments on the basis of *nifH* gene amplicons and metagenomics [32, 34]. These diazotrophs are thought to depend on reduced organic compounds for their energy and carbon sources [35]. However, more studies have shown that some chemolithoautotrophic bacteria occupy a relative high abundance in mangrove sediment ecosystems [36, 37]. For example, members of the genera *Sulfurovum*, *Sulfurimonas*, *Thermodesulfovibrio*, *Desulfobacterium*, and *Desulfococcus* are abundant, with relative abundances >1% in the sediments (0–20 cm) of Yunxiao mangroves [36]. In other mangrove sediments collected from six locations along the coastline of the BeibuGulf in Guangxi Province, China, chemolithoautotrophic taxa such as *Desulfococcus*, *Nitrosopumilus*, and *Sulfurimonas* are also predominant [38]. The dominance of chemolithoautotrophs in such organic-rich environments raises the question of whether the nitrogen fixation process is mediated by these autotrophs. Considering that mangrove sediment ecosystems are rich in reduced inorganic compounds such as H_2_ and sulfide produced by the degradation of organic matter [39, 40], we hypothesize that the oxidation of these reduced compounds is another crucial energy pathway for nitrogen fixation.

In this study, to address the roles of chemolithoautotrophic diazotrophs in organic-enriched sediments, we quantified the concentrations of carbon, nitrogen, and sulfur in sediments, carried out activity measurements, and applied metagenomics and metatranscriptomics to investigate nitrogen-fixing microorganisms in mangrove sediments. Furthermore, we inferred the potential metabolic capabilities of the dominant chemolithoautotrophic diazotrophs and confirmed their nitrogen-fixing capacity using culture-dependent methods. Overall, this study reveals the true diazotrophic populations in mangrove surface sediments, and sheds new light on the significant role of chemolithoautotrophs in dark nitrogen fixation within mangrove ecosystems.

## Materials and methods

### Site description and sampling

The sampling site is located in the mangrove wetland of Jiulong River tributaries in Zhangzhou (117° 45′ N, 24° 20′ E), Fujian Province, China (See online supplementary material for a colour version of Fig. S1), with a mean annual temperature of 21.2°C and an annual precipitation of 1714.5 mm. The irregular semidiurnal tides were on average 7.70 and − 3.03 m of high and low tide levels, respectively. Sediment cores were collected in August 2022 using a 20-cm long PVC sampling column after ebb and sliced at 2-cm intervals into 10 layers (0–2, 2–4, 4–6, 6–8, 8–10, 10–12, 12–14, 14–16, 16–18, and 18–20 cm), yielding a total of 10 samples. At the sampling site, the sediments were not invaded by mangrove roots, and there was no apparent bioturbation. Sediment colors served as an indicator for the presence of active sulfide including the uppermost layer (brownish, sulfide-free), sulfide transition zone (brown to gray), or sulfidic layer (gray or dark, sulfide-rich). Sliced sediments were stored in a portable cooler at 4°C and transported back to the laboratory within 24 h. Each sample was then divided into two subsamples: one was stored at 4°C for physicochemical properties analysis, and the other was kept at −80°C for DNA and RNA extraction.

### Physicochemical properties analysis

The water content of sediments was measured by drying 10.0 g of fresh sediment at 105°C to a constant weight [34]. The pH and salinity of the sediments were measured in suspensions containing 2.0 g dry sediment in a 1:2.5 (sediment/water) ratio and 1:5 (sediment/water) ratio with a pH meter (SevenCompact S220, Mettler Toledo, OH, USA) and a salinity meter (Abbemat 300, Anton Paar, Graz, Austria), respectively [37]. Redox potential was measured using a digital voltmeter (Abbemat 550, Anton Paar, Graz, Austria) with Pt and Ag/AgCl reference electrodes [41]. Sediment ammonium (NH_4_^+^), nitrate (NO_3_^−^), and nitrite (NO_2_^−^) were extracted using 2 M KCl, and measured with a continuous flow auto-analyzer (AA3, Bran–Luebbe, Hamburg, Germany) [42]. Porewater sulfate (SO_4_^2−^) and thiosulfate (S_2_O_3_^2−^) concentrations were measured in porewater extracted from 10.0 g of fresh sediment by an ion chromatography (Dionex ICS-1100, Thermo Scientific, MA, USA) [43]. Acid volatile sulfide (AVS) was treated with acid to release H_2_S and measured by the iodometric titration method [44]. Sediment samples for measuring the total carbon (TC), total nitrogen (TN), and total sulfur (TS) were dried at 65°C to a constant weight, finely ground, and then measured by a Flash 2000 CHNS/O elemental analyzer (Thermo Scientific, MA, USA) [45]. Total organic carbon (TOC) was measured using the same elemental analyzer after the samples were digested with 5% HCl [19].

### DNA extraction and 16S rRNA gene amplicon sequencing

A total of 10 sediment samples (5.0 g) were subjected to DNA extraction with a DNeasy PowerMax Soil Kit (12988–10, QIAGEN, Hilden, Germany) according to the manufacturer’s protocol. Quality assessment was achieved using a NanoPhotometer spectrophotometer (IMPLEN, CA, USA) and a Qubit 2.0 Fluorometer (Life Technologies, CA, USA). The V3-V4 region of the bacterial 16S rRNA gene was amplified with the universal primers 338F and 806R [46]. Amplicon sequencing was performed on a MiSeq platform (Illumina) using 2 × 300 bp chemistry. Reads were quality controlled using fastp (v0.19.6) [47] and then merged using FLASH (v1.2.11) [48]. The amplicon sequence variant (ASV) was obtained after denoising and removal of chimeras by DADA2 [49] algorithm recommended by QIIME2 [50], and classified using a naive Bayesian classifier in QIIME2 (feature-classifier classify-sklearn) with a confidence score of 0.7 (—p-confidence) against the SILVA v138 database [51].

### Quantitative PCR analysis of 16S rRNA gene

Quantitative PCR (qPCR) analyses were performed to estimate the abundance of bacteria and archaea at different depths in the sediment core. PCR reactions were set up using Bio-Rad SsoAdvanced Universal SYBR Green Supermix under the following conditions: 98°C for 2 min, 30 cycles of 98°C for 30 s, 50°C for 30 s, and 72°C for 1 min. Amplification of bacterial and archaeal 16S rRNA genes was performed with the domain-specific primers 338F-806R and 524F10extF-Arch958RmodR, respectively [52]. The specificity of the amplified products was confirmed by melting curve analysis and gel electrophoresis. Standards with known 16S rRNA gene copy numbers were serially diluted from 6.03 × 10^10^ to 6.03 × 10^3^ copies/μl, and the amplification efficiency was between 90% and 105%. The bacterial and archaeal community abundances are shown in, See online supplementary material for a colour version of, Fig. S2.

### Metagenomic sequencing, assembly, and binning

The metagenomic DNA of 10 sediment samples was extracted using DNeasy PowerMax Soil Kit as described above. DNA library was prepared with the NEBNext UltraTM DNA Library Prep Kit (E7645, New England Biolabs, MA, USA) following the manufacturer’s protocols. The libraries were then measured using an Agilent 5300 Bioanalyzer (Agilent Technologies, CA, USA), and quantification was done using real-time PCR. Sequencing was performed on the HiSeq 2500 platform (Illumina) with 2 × 150 bp paired-end reads run at Majorbio Biotechnology Co. Ltd., (Shanghai, China). Raw reads were filtered, quality controlled, and trimmed using fastp v0.23.2 with default parameters [47]. All clean reads from different samples were individually assembled with MEGAHIT (v1.1.3) [53] with default settings. Genes were predicted for CDSs on assembled contigs with Prodigal v2.6.3 with the -p meta option [54]. These sequences were then clustered at 95% amino acid identity (AAI) using CD-HIT (v4.8.1) [55] with the parameters: -c 0.95 -aS 0.9 -g 1 -d 0, which yielded a total of 6 599 066 non-redundant gene clusters.

Assembled contigs were filtered by length (>1000 bp) for subsequent binning. Each metagenomic assembly was binned using the metaWRAP v1.3.2 binning module (parameters: -maxbin2 -concoct -metabat2) [56]. All individual assemblies were also concatenated and binned separately using the VAMB tool (v3.0.2; parameters: —minfasta 200 000 -o C) [57]. The produced bins from each binning tool were integrated and refined using the Bin_refinement module of metaWRAP (v1.3.2; parameters: -c 50 -x 10). All produced bins were aggregated and dereplicated to a non-redundant set of strain-level metagenome-assembled genomes (MAGs) using dRep v3.4.0 (parameters: -comp 50 -con 10) [58] at 95% average nucleotide identities. Completeness, contamination, and heterogeneity of MAGs were evaluated using CheckM v1.2.1 [59]. Additionally, we used GUNC (v1.0.5; default parameters) [60] to assess chimerism and contamination of all diazotrophic MAGs, and MAGpurify software (v2.1.1; default parameters) [61] to check the potential misassigned contigs based on the phylo-markers, clade-markers, tetra-freq, gc-content, and clean-bin modules.

### Taxonomic classification of MAGs

Taxonomic annotations of each MAG were initially performed using GTDB-Tk v2.4.0 with the “classify_wf” workflow (default parameters) against the reference database (R220) [62]. The assignments were confirmed by the visual inspection of taxonomic trees. Reference genomes downloaded from NCBI GenBank and MAGs from this study were used to construct a phylogenomic tree based on the concatenation of 43 conserved single-copy genes extracted by CheckM v1.2.1. The maximum-likelihood phylogenomic tree was constructed using IQ-TREE (v2.2.0.3) [63] with the “-m MFP -B 1000” options. All produced phylogenomic trees were visualized using Interactive Tree of Life (iTOL, v5) [64].

### Functional annotation

For functional profiling of the non-redundant gene catalog, we used the pipeline of Greening lab metabolic marker gene database v.1 with DIAMOND v0.9.14 [65, 66]. Searches were carried out using all quality-filtered unassembled reads with lengths over 140 bp. These genes were involved in sulfur cycling (*fcc*, *sqr*, *soxB*), nitrogen cycling (*nifH*), carbon fixation (*aclB*, *rbcL*, *acsB*), and NiFe-hydrogenases. Results were filtered based on an identity threshold of 50%, except for NifH and AcsB (65%), and NiFe-hydrogenases (60%) [67]. For individual MAGs and genomes from isolates, gene prediction was performed using Prodigal (v2.6.3, default settings), and the predicted genes were further annotated using KEGG Automatic Annotation Server [68], KEGG-Decoder [69], METABOLIC v4.0101 [70], and Rapid Annotation Using Subsystems Technology approach (RAST, v2.0) [71].

### Phylogenetic analyses and conserved residues of functional genes

For each gene, amino acid sequences from the current study were aligned with reference sequences using MAFFT (v7.490, −auto option) [72], and trimmed using trimAl (v1.2.59, −gappyout option) [73]. Maximum likelihood phylogenetic trees were constructed using IQ-TREE (v2.2.0.3) with best-fit models and 1000 ultrafast bootstraps. All the tree files were visualized and embellished with iTOL v5. Multiple alignment of NifH, NifD, and NifK superfamily sequences for conserved active site residue analysis was performed using MAFFT (EMBL-EBI) [74] and visualized with Jalview v2.11.2.0 [75].

### Abundance profiles

At the contig level, relative abundances of *nifH* gene in 10 metagenomes were calculated from non-redundant gene catalog using the program Salmon (v1.9.0) [76] in the mapping-based mode (parameters: -validate Mappings -meta). Genes per million (GPM) values were used as a proxy for gene abundance, as described elsewhere [77]. GPM value was normalized based on the gene length and sequencing depth [78]. At the genome level, the relative abundance of each *nif*-containing MAG was profiled by mapping quality-trimmed reads from the 10 metagenomes against the MAGs using CoverM v1.2.1 in genome mode (parameters: -m relative_abundance —trim-min 0.10 —trim-max 0.90 —min-read-percent-identity 0.95 —min-read-aligned-percent 0.75 —min-covered-fraction 0) [79].

### Metatranscriptomic analysis

Total RNA was extracted from the same samples used for metagenome analysis using the RNeasy PowerSoil Total RNA Kit (12866–25, QIAGEN, Hilden, Germany) according to the manufacturer’s protocol. RNA purity and concentration were evaluated using a Qubit 2.0 Fluorometer (Life Technologies, CA, USA). RNA integrity was determined using an Agilent 5300 Bioanalyzer (Agilent Technologies, CA, USA). Whole transcriptome amplification of total RNA was carried out using the RNA REPLI-g Cell WGA & WTA Kit (150054c, QIAGEN, Hilden, Germany) according to the manufacturer’s protocol. To enrich messenger RNA (mRNA), ribosomal RNA was depleted from total RNA using the RiboCop rRNA Depletion Kit (Lexogen, Vienna, Austria). Whole mRNA-Seq libraries were generated by Majorbio Biotechnology Co. Ltd., (Shanghai, China) using the NEBNext Ultra Nondirectional RNA Library Prep Kit (E6111, New England Biolabs, MA, USA) following the manufacturer’s instructions. The constructed libraries were sequenced on a NovaSeq 6000 platform (Illumina), and 150 bp paired-end reads were generated.

Raw metatranscriptomic reads were quality filtered in the same manner as metagenomes. The reads corresponding to ribosomal RNAs were removed using SortMeRNA v.4.3.4 [80] with default parameters with the smr_v4.3_default_db database. Subsequently, these high-quality metatranscriptomic reads were mapped to predicted protein-coding genes from the reference gene catalog and *nifH*-containing MAGs using Salmon v.1.9.0 [76] in mapping-based mode (parameters: -validate Mappings -meta), with mapping rates of 7.11%–14.87% and 0.02%–0.67%, respectively. The expression level of each gene was normalized to transcripts per million (TPM) based on gene length and sequencing depth.

### Pure culture isolation and growth characteristics

Sediment samples were collected in January 2019 from a mangrove wetland in Zhangzhou as described above. For isolation, 1.0 g sediment samples were transferred into 50 ml serum bottles containing 10 ml MMJS medium with a gas phase mixture of 80% N_2_/18% CO_2_/2% O_2_ (200 kPa), and incubated at 28°C as previously described [81]. Cells were purified via the dilution-to-extinction technique using the same medium. The purity of the culture was confirmed by microscopic examination and 16S rRNA gene sequencing. Genomic DNA of pure cultures was extracted using the method described by Jiang *et al*. (2009) [82] and sequenced by Majorbio Biotechnology Co. Ltd., (Shanghai, China) using a HiSeq 4000 platform (Illumina, San Diego, CA, USA).

Heterotrophic growth was tested in a MMJS medium without NaHCO_3_ with a series of organic compounds as the sole carbon source under a gas phase of 76% N_2_/20% CO_2_/4% O_2_ (200 kPa) [83]. These organic carbon sources included: 0.1% (w/v) peptone, yeast extract, tryptone, starch, casein, and casamino acids, 5 mM of acetate, formate, citrate, tartrate, succinate, propionate, and pyruvate, 5 mM each of 20 amino acids, 0.02% (w/v) sucrose, galactose, glucose, lactose, fructose, maltose, and trehalose. The utilization of these organic compounds as alternative energy sources was also examined in MMJ medium in the absence of thiosulfate under a gas phase of 76% N_2_/20% CO_2_/4% O_2_ (200 kPa). Additionally, to examine the utilization of inorganic and organic nitrogen sources, ammonium chloride (1 mM), sodium nitrate (1 mM), sodium nitrite (1 mM), urea (1 mM), or a mixture of 20 amino acids (1 mM) was added to MMJHS medium lacking all nitrogen sources under a gas phase of 76% H_2_/20% CO_2_/4% O_2_ (200 kPa).

### Characterization of N_2_ fixation in mangrove sediment samples and isolates

An acetylene reduction assay with slight modifications was used to measure nitrogenase activity [84, 85]. Approximately 1.0 g sediment samples with four layers (0–2, 6–8, 12–14, and 16–18 cm) were separately mixed with 10 ml autoclaved MMJ medium in a 60 ml serum bottle with 10% (v/v) acetylene added to the headspace. Autotrophic N_2_-fixing potential was tested with 10 mM Na_2_S_2_O_3_ as the energy source and 10 mM NaHCO_3_ as the carbon source under 70% H_2_/20% CO_2_ (200 kPa). For heterotrophic diazotrophic potential, 10 mM sucrose was supplied as the carbon and energy sources. A blank control treatment was set up by adding only sediments. For the 0–2 cm layer, microoxic condition was set with 4% O_2_ as the sole electron acceptor during incubation. For the other three layers, S^0^ (5 g L^−1^) was added for anoxic condition, and sulfide was produced with a smell like rotten eggs. Samples were incubated at 30°C and 180 rpm in the dark. A 7890B GC-FID (Agilent Technologies, CA, USA) equipped with an Al/KCl capillary column (Agilent Technologies, CA, USA) was used to monitor the production of ethylene in the headspace.

The ^15^N activity of the sediments and isolates was determined using the ^15^N_2_ assimilation method with slight modifications (e.g. time and volume) [19]. The culture conditions and treatment sets were established as described above. All cultures were incubated at 30°C in the dark and analyzed on Day 10 for sediments and Day 1 for isolates. All samples including sediments and isolates were then harvested by centrifugation (10 000 × g, 4°C, 20 min) after turbidity became apparent, washed twice in cold 20 mM Tris buffer in artificial sea water, and freeze dried overnight [86]. The atomic % ^15^N of freeze-dried cells was determined using a Carlo-Erba elemental analyzer (Model NA 1500, Fisons Instruments, MA, USA) linked to a Finnegan MAT (ThermoQuest, CA, USA) Delta S isotope ratio mass spectrometer [22].

## Results and discussion

### Physicochemical characteristics of mangrove sediments

We measured TC, TOC, TN, NO_3_^−^, NO_2_^−^, NH_4_^+^, TS, AVS, SO_4_^2−^, redox potential, moisture, pH, and salinity to characterize their vertical distributions from 0 to 20 cm depth in Zhangzhou mangrove sediments (See online supplementary material for a color version of Fig. S3). High concentrations of TC and TOC (2.41%–2.59% and 2.10%–2.35%, respectively) were observed at all depths, whereas TN concentrations were low, ranging from 0.12% to 0.14% (See online supplementary material for a color version of Fig. S3 A-C). The C/N ratio ranged from 18.39 to 21.97 in the 0–20 cm layer, which is similar to that in other mangrove sediment ecosystems, and distinct from that in other habitats, such as tidal flats, brackish water, and freshwater [87, 88]. The concentrations of inorganic nitrogen, including NO_3_^−^ (0.14–0.65 mg/kg) and NO_2_^−^ (0.01–0.04 mg/kg), were low and decreased consistently with depth (See online supplementary material for a color version of Fig. S3 D and E). NH_4_^+^ concentrations varied between 1.27 and 3.23 mg/kg at all sediment depths (See online supplementary material for a color version of Fig. S3 F). Similar low ammonium-N concentrations have been reported in other mangrove habitats [34, 36, 89], underscoring the nitrogen limitation in Zhangzhou mangrove surface sediments.

High concentrations of TS and AVS increased with sediment depth, peaking in the 18–20 cm layer (See online supplementary material for a color version of Fig. S3 G and H), whereas the SO_4_^2−^ concentration exhibited a decreasing trend, with the highest concentration in the upper 0–6 cm layer (See online supplementary material for a color version of Fig. S3 I). Traces of thiosulfate (S_2_O_3_^2−^) were detected only in surface sediments (~40 and ~ 20 μM in the 0–2 and 2–4 cm layers, respectively). The redox potential (Eh) of the top 0–2 and 2–4 cm layers was 320 and 252 mV, respectively, and this value decreased sharply with depth below 4 cm, reaching the lowest value of −120 mV at the deepest layer (See online supplementary material for a color version of Fig. S3 J). Among all measured parameters, TN, NO_3_^−^, NO_2_^−^, and SO_4_^2−^ were negatively correlated with depth (*P* < .05), whereas TS and AVS showed a positive correlation (*P* < .05) (See online supplementary material for a color version of Fig. S4). Collectively, these findings highlight that Zhangzhou mangrove surface sediments are characterized by a carbon and nitrogen imbalance coupled with a richness of reduced sulfur compounds.

### Diversity, distribution, and activity of nitrogen-fixing genes across sediment depths

**Figure 1:**
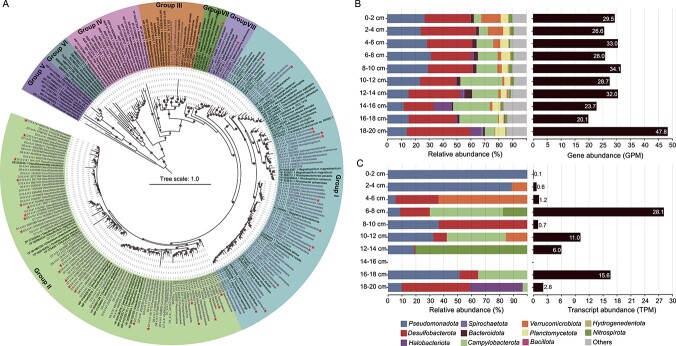
**Summary of 154 non-redundant homologs of the nitrogenase subunit NifH from mangrove sediments.** A, Maximum-likelihood phylogenetic tree of NifH amino acid sequences. *nifH* homologs were classified into canonical groups I to III, nitrogen fixation-like groups IV to VI, and newly assigned groups VII and VIII. The NifH sequences obtained in this study are highlighted, and sequences from diazotrophic MAGs are marked by stars. The scale bar indicates the mean number of substitutions per site. B, Relative abundances of *nifH* genes affiliated with different taxonomic groups (left) and the total values of gene abundance (right) in different mangrove sediment samples. C, Relative transcript abundances of *nifH* genes affiliated with different taxonomic groups (left) and the total values of transcript expression (right) in different sediment samples. The “others” category represents unassigned sequences.

The *nifH* gene, encoding an essential nitrogenase enzyme protein, is commonly used to investigate the diversity and prevalence of diazotrophs across diverse settings [90, 91]. Annotations of contigs assembled from the 10 metagenomes extracted from the mangrove sediment samples revealed a total of 154 non-redundant *nifH* homologs falling into the nitrogenase superfamily (Fig. 1A; Table S1). Except for nitrogenase-like groups of IV to VI [92], these homologs were classified into canonical nitrogenase sequences (groups I-III) [93] and two newly proposed lineages, groups VII and VIII^10^ (Fig. 1A). Two novel lineages are also considered to be bona fide *nifH* based on the analyses of nitrogenase conserved motifs, as detailed below. These results indicate that the mangrove sediments host more diverse nitrogenase genes than previously thought [34].

The abundances of *nifH* gene ranged from 20.1 to 34.1 GPM from the surface to 18 cm, peaking at 47.8 GPM in the 18–20 cm layer (Fig. 1B). At the phylum level, the most abundant putative diazotrophs in the mangrove sediments were *Desulfobacterota*, *Pseudomonadota*, and *Campylobacterota* (Fig. 1B). At the class level, *Gammaproteobacteria*, *Campylobacteria*, and *Desulfobacteria* were the most prevalent (See online supplementary material for a color version of Fig. S5A). Despite the absence of transcripts in the 14–16 cm layer, *nifH* transcripts were detected throughout the other layers, indicating that nitrogen-fixing activity is present at most sediment depths (Fig. 1C). At the four layers with high *nifH* transcripts, *Campylobacterota*, *Nitrospirota*, and *Pseudomonadota* emerged as the potential predominant nitrogen-fixing groups (Fig. 1C). Specifically, there was a niche differentiation among these groups. In the 6–8 and 10–12 cm layers, *nifH* transcripts values were mainly affiliated with the members of the class *Campylobacteria* (See online supplementary material for a color version of Fig. S5B), and in the 12–14 cm layer, *nifH* transcripts were highly expressed in the class *Thermodesulfovibrionia*. In the 16–18 cm layer, higher *nifH* transcripts values were found from the classes *Gammaproteobacteria* and *Campylobacteria* (See online supplementary material for a color version of Fig. S5B).

Considering the dominant and transcriptionally active diazotrophs were mainly belonged to the chemolithoautotrophic *Campylobacterota* and *Nitrospirota*, further exploration was conducted to determine the correlation between *nifH* and carbon fixation genes (e.g. *aclB*, *rbcL*, and *acsB*), sulfur oxidation genes (e.g. *sqr* and *soxB*) or hydrogen oxidation gene (*hydB*) within mangrove sediments. The results revealed strong and significant correlations between the transcriptional activity of *nifH* and the aforementioned genes (See online supplementary material for a color version of Fig. S6), suggesting a robust link between diazotrophic and putative chemolithoautotrophic populations in mangrove sediments. Therefore, it is assumed that these chemolithoautotrophic microorganisms may constitute key active groups for *in situ* nitrogen fixation in mangrove sediments.

### Potential nitrogen-fixing microorganisms identified within mangrove sediments

**Figure 2:**
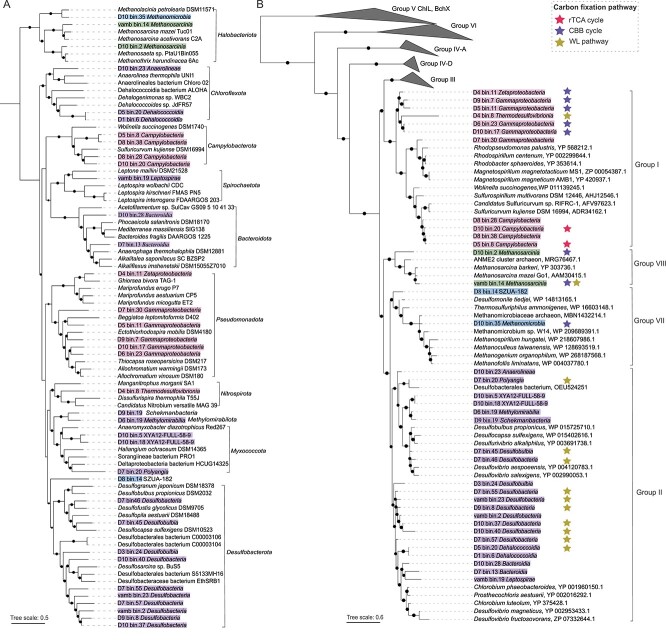
**Phylogenetic trees of nitrogen-fixing MAGs and their NifH protein sequences.** A, Maximum-likelihood phylogenetic tree of 36 MAGs containing nitrogen fixation genes based on the concatenation of 43 conserved protein sequences. MAGs are color-coded according to their NifH homolog groups at the phylum level. B, Maximum-likelihood phylogenetic tree of identified NifH protein sequences within 36 MAGs. Stars of different colors indicate different carbon fixation pathways. For both trees, bootstrap values greater than 50% are denoted at the nodes, and scale bars represent the average number of substitutions per site.

Through metagenomic assembly and binning strategies, we recovered 180 bacterial (*n* = 172) and archaeal (*n* = 8) population genomes with >50% completeness and < 10% contamination, which belonged to 24 phyla based on the GTDB taxonomy (Table S2). Among these genomes, 36 MAGs (33 bacterial and 3 archaeal MAGs) spanning 12 phyla were identified as potential nitrogen-fixing microorganisms, including *Desulfobacterota* (*n* = 10), *Pseudomonadota* (*n* = 6), *Campylobacterota* (*n* = 4), *Chloroflexota* (*n* = 3), *Halobacteriota* (*n* = 3), *Myxococcota* (*n* = 3), *Bacteroidota* (*n* = 2), *Methylomirabilota* (*n* = 1), *Nitrospirota* (*n* = 1), *Spirochaetota* (*n* = 1), *Schekmanbacteria* (*n* = 1), and SZUA-182 (*n* = 1) (Fig. 2A; Table S3). Subsequent phylogenetic analyses revealed that nitrogenases from these 36 MAGs were categorized into groups I, II, VII, and VIII (Fig. 2B), and all of which were observed to have conserved active sites among NifH, NifD, and NifK (See online supplementary material for a color version of Figs. S7–S9). Furthermore, NifH sequences from the same taxonomic group did not cluster into a single clade (Fig. 2A and B), which may be explained by horizontal gene transfer [94, 95].

Among 36 potential nitrogen-fixing MAGs, a majority (21/36) encoded carbon fixation pathways, with at least 60% of the genes and all the key enzymes [96] (Fig. 2B and See online supplementary material for a color version of Fig. S10). The reductive citric acid (rTCA) cycle was encoded by the class *Campylobacteria* (*n* = 2) of the phylum *Campylobacterota* (Fig. 2B and See online supplementary material for a color version of Fig. S11). The Calvi–Benson–Bassham (CBB) cycle was encoded by five *Pseudomonadota* MAGs including the classes *Gammaproteobacteria* (*n* = 4) and *Zetaproteobacteria* (*n* = 1), and three *Halobacteriota* MAGs including the classes *Methanosarcinia* (*n* = 2) and *Methanomicrobia* (*n* = 1) (Fig. 2B and See online supplementary material for a color version of Fig. S12). The Wood–Ljungdahl (WL) pathway was encoded by eight *Desulfobacterota* MAGs including the classes *Desulfobacteria* (*n* = 6) and *Desulfobulbia* (*n* = 2), one *Myxococcota* MAG including the class *Polyangia* (*n* = 1), one *Nitrospirota* MAG including the class *Thermodesulfovibrionia* (*n* = 1), and one *Halobacteriota* MAG including the class *Methanosarcinia* (*n* = 1) (Fig. 2B and See online supplementary material for a color version of Fig. S13). These findings suggest that the microorganisms represented by these MAGs could be potential chemolithoautotrophic diazotrophs.

**Figure 3:**
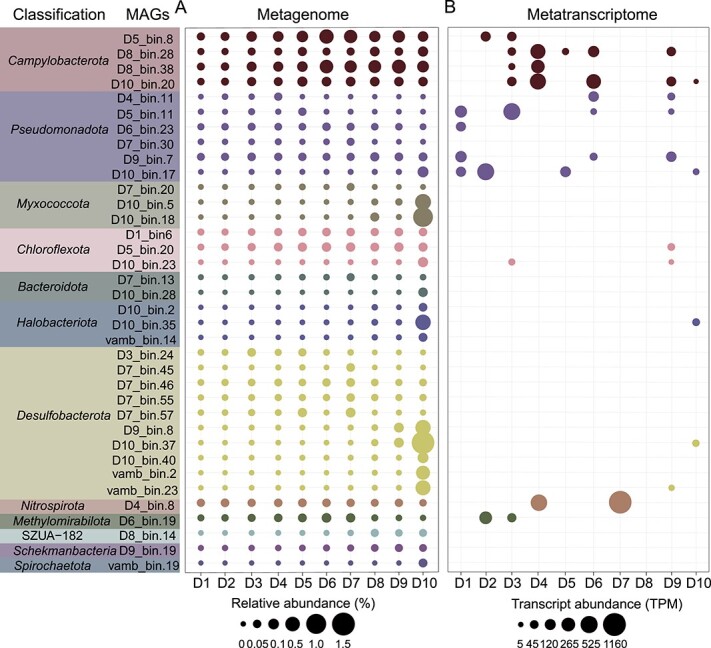
**Relative abundance and expression of transcripts for 36 nitrogen-fixing MAGs at different mangrove sediment depths.** A, The relative abundance of each MAG was estimated using CoverM. B, The expression of transcripts for each MAG is represented in units of transcripts per million (TPM). The detailed information is given in Table S4 and Table S5.

Read mapping of the 36 diazotrophs showed that they were widely distributed at different sediment depths (Fig. 3A and Table S4). When considered individually, the chemolithoautotrophic taxon *Campylobacterota* was the most abundant at most sediment depths, except 18–20 cm layer (Fig. 3A), indicating its important role as a potential nitrogen fixer in mangrove sediments. A previous study indicated that *Campylobacteria* was abundant in the surface (0–15 cm) mangrove sediments, coupling sulfur oxidation and denitrification processes [36]. Furthermore, *Pseudomonadota* and chemolithoautotrophic *Nitrospirota* also exhibited higher abundances in the 0–18 cm layer, but demonstrated a consistent decrease in abundance with sediment depth (Fig. 3A). Whereas in the 18–20 cm layer, members of the phyla *Desulfobacterota*, *Myxococcota*, and *Halobacteriota* were the most predominant diazotrophs (Fig. 3A). The high abundances could point to an important role for these phyla in nitrogen fixation in deeper sediments. A recent study indicated that in deeper mangrove sediments ~30 cm, members from *Desulfobacterota* and *Halobacteriota* were dominant [37]. At the class level, *Campylobacteria*, followed by several other classes including *Desulfobacteria*, *Gammaproteobacteria*, *Dehalococcoidia*, and *Thermodesulfovibrionia*, were notably predominant in the 0–18 cm layer, whereas in the 18–20 cm layer, the classes such as *Desulfobacteria*, YA12-FULL-58-9, and *Methanomicrobia* were the most abundant (See online supplementary material for a color version of Fig. S14A). Therefore, chemolithoautotrophic organisms consistently emerge as the dominant nitrogen fixers in the top 0–20 cm of mangrove sediments.

To evaluate the *in situ* expression of these diazotrophs, 10 metatranscriptomes from different sediment depths were mapped against MAGs encoding nitrogenase (Table S5). The results showed that *nifH* gene transcripts in the 0–18 cm layer were predominantly expressed in 10 chemolithoautotrophic MAGs from the phyla *Campylobacterota* (*n* = 4), *Pseudomonadota* (*n* = 5), and *Nitrospirota* (*n* = 1) (Fig. 3B), suggesting that they could be the primary nitrogen fixers in mangrove sediments. At the class level, the *nifH* genes of *Campylobacteria* were transcribed from low to high levels in several layers, up to 1456.38 TPM, and the transcript values from *Thermodesulfovibrionia* in the 6–8 and 12–14 cm layers were 412.15 and 1122.25 TPM, respectively (See online supplementary material for a color version of Fig. S14B). Furthermore, *nifH* transcripts were expressed in the class *Gammaproteobacteria* in multiple sediment layers with higher levels in the surface layers (0–6 cm), whereas this transcription was found in *Zetaproteobacteria* in the 10–12 cm and 16–18 cm layers, with 135.17 and 41.94 TPM, respectively (See online supplementary material for a color version of Fig. S14B). This study is the first report indicating that *Zetaproteobacteria* and *Thermodesulfovibrionia* can actively fix nitrogen, implying that they may play important roles in the nitrogen cycle in addition to their previously reported roles [96, 97]. In the deepest layer (18–20 cm), the *nifH* transcripts were mainly expressed in the phyla *Desulfobacterota* and *Halobacteriota,* but with lower values of 10.05–30.82 and 30.30 TPM, respectively (Fig. 3B), indicating that these taxa may play elevated roles in nitrogen fixation in deeper layers. Interestingly, no *nifH* transcripts from previously reported heterotrophic diazotrophs [52, 98, 99], such as certain MAGs from the phyla *Myxococcota* (*n* = 2), *Bacteroidota* (*n* = 2), or *Spirochaetota* (*n* = 1), were expressed (Fig. 3B). Overall, our results further confirm that chemolithoautotrophs dominate dark nitrogen fixation in mangrove sediments.

### Energy production pathways of dominant chemolithoautotrophic diazotrophs

**Figure 4:**
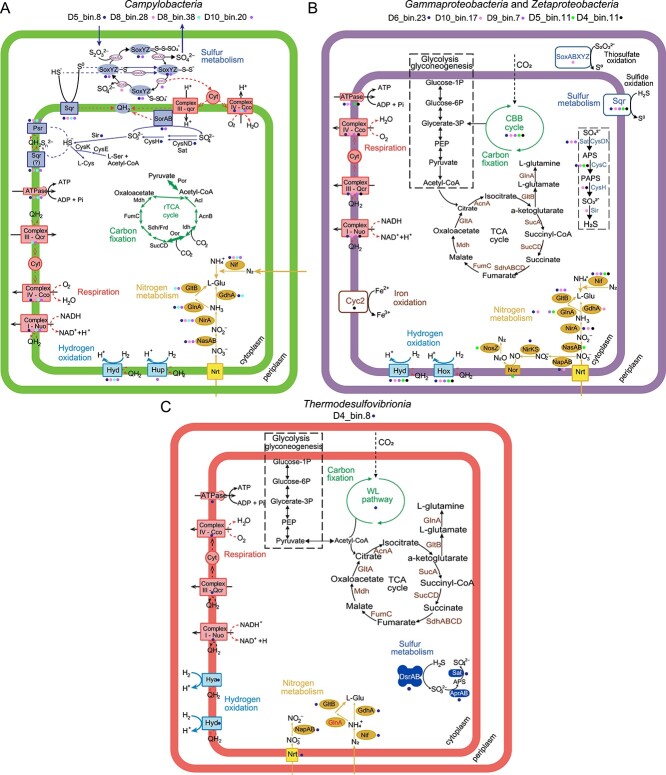
**Metabolic pathway reconstructions for MAGs of dominant nitrogen-fixing chemolithoautotrophs.** Metabolic pathways are inferred for *Campylobacterota* (A), *Pseudomonadota* (B), and *Nitrospirota* (C), with the carbon fixation pathways of rTCA, CBB, and WL, respectively. Steps with more than one arrow indicate that several operons encoding different enzymes and catalyzing that reaction are present in the genome. Enzymes that are absent within these reconstructions are highlighted. Comprehensive enzyme annotations are provided in Tables S6-S8.

To predict the functional capabilities of dominant chemolithoautotrophic diazotrophs, the metabolic potentials of MAGs were determined based on marker genes and pathways (Fig. 4A; Tables S6-S8). For sulfur metabolism, all *Campylobacterota* MAGs (D5_bin.8, D8_bin.38, D8_bin.28, and D10_bin.20) harbored multiple copies of genes encoding sulfide:quinone oxidoreductase (Sqr) (Fig. 4A and See online supplementary material for a color version of Fig. S15), which catalyzes the oxidation of sulfide to elemental sulfur [100]. A nearly complete Sox system (SoxACDXYZ) was identified in D10_bin.20, indicating that it has the genetic potential to oxidize thiosulfate [101]. D5_bin.8 and D8_bin.28 contained partial Sox systems (Fig. 4A and See online supplementary material for a color version of Fig. S16), potentially due to the low MAG completeness. Moreover, D5_bin.8 and D10_bin.20 contained genes encoding sulfite dehydrogenase (SorAB), indicating that they can oxidize sulfite to sulfate [101]. For hydrogen metabolism, all four MAGs contained Group 1 [NiFe]-hydrogenases (Hyd and Hya), and only D10_bin.20 contained Group 2 [NiFe]-hydrogenases (Hup) (Fig. 4A and See online supplementary material for a color version of Fig. S17), which may endow them the potential to use hydrogen as an energy source [102]. Regarding electron acceptors, D5_bin.8, D8_bin.38, and D10_bin.20 contained oxygen-utilizing genes encoding *cbb3*-type cytochrome c oxidases (CcoNOPQ) and *caa3*-type cytochrome c oxidases (CoxAB) (Fig. 4A). In comparison with the low-oxygen-affinity *caa3*-type oxidase induced under oxic conditions, *cbb3*-type oxidase is a high-affinity terminal oxygen reductase capable of functioning under microoxic to anoxic conditions [103, 104]. The presence of cytochromes could also function as residual O_2_ scavengers for the detoxification of O_2_/reactive oxygen species to protect O_2_-sensitive proteins [105, 106]. Additionally, polysulfide reductase (Psr), which is involved in elemental sulfur reduction, was encoded by four MAGs, indicating their potential ability to perform sulfur reduction under anoxic conditions [100]. These results show that these *Campylobacteria* can use reduced sulfur compounds and hydrogen as electron donors, and oxygen and elemental sulfur as terminal electron acceptors to generate ATP for nitrogen fixation.

Within the phylum *Pseudomonadota*, four MAGs (D5_bin.11, D6_bin.23, D9_bin.7, D10_bin.17) belonged to the class *Gammaproteobacteria*, and one MAG (D4_bin.11) belonged to the class *Zetaproteobacteria* (Table S3). For sulfur metabolism, all MAGs harbored different copies of *sqr* for sulfide oxidation and lacked SorAB for sulfite oxidation (Fig. 4B and See online supplementary material for a color version of Fig. S15). D5_bin.11 and D10_bin.17 encoded an incomplete Sox system (SoxABXYZ), and D6_bin.23 only contained the subunits of SoxAXYZ (Fig. 4B and See online supplementary material for a color version of Fig. S16). For hydrogen metabolism, all MAGs encoded Group 1 [NiFe]-hydrogenase (Hdy and/or Hya) and lacked Group 2 [NiFe]-hydrogenase (Hup) (Fig. 4B and See online supplementary material for a color version of Fig. S17). The gene encoding cytochrome *c*-porin (Cyc2), which is involved in iron oxidation, was found in D4_bin.11. *Zetaproteobacteria* are known to be obligate chemolithoautotrophic iron-oxidizing bacteria that oxidize Fe at a circumneutral pH [97]. With respect to electron acceptors, D5_bin.11, D6_bin.23, and D10_bin.17 contained the genes encoding CcoNOPQ, CoxAB, and a cytochrome *bd* ubiquinol oxidase (CydAB) for oxygen respiration, which has a high affinity for oxygen and allows growth under microoxic conditions [102]. D9_bin.7 encoded genes for CcoNOPQ and CydA, and D4_bin.11 encoded genes for CcoNOPQ and CoxAB. Furthermore, D6_bin.23 and D10_bin.17 contained NapAB for nitrate reduction to nitrite, and only NorBC and NosZ were found in D5_bin.11 (Fig. 4B). Furthermore, Psr involved in elemental sulfur reduction was encoded by most *Gammaproteobacteria* MAGs. These results show that *Gammaproteobacteria* and *Zetaproteobacteria* can use sulfide, thiosulfate, iron, or hydrogen as electron donors, and oxygen, nitrate, and elemental sulfur as terminal electron acceptors to generate ATP for nitrogen fixation.

One MAG (D4_bin.8) belonged to the class *Thermodesulfovibrionia* from the phylum *Nitrospirota* (Table S4). For sulfur metabolism, D4_bin.8 lacked all genes encoding sulfur oxidation such as Sqr, Sox, and Sor (Fig. 4C). Furthermore, D4_bin.8 encoded Group 1 [NiFe]-hydrogenase (Hyd and Hya) and NAD-reducing hydrogenase (HoxHYU) for hydrogen oxidation (Fig. 4C and See online supplementary material for a color version of Fig. S17). With respect to electron acceptors, D4_bin.8 contained all genes encoding the sulfate reduction pathway, including dissimilatory sulfite reductase (DsrABC) and adenylylsulfate reductase (AprAB) (Fig. 4C). D4_bin.8 also contained the enzymes CcoNOPQ and CoxAB for oxygen respiration and NapAB for nitrate reduction to nitrite. The Psr for elemental sulfur reduction was absent in D4_bin.8. These results show that *Thermodesulfovibrionia* can couple hydrogen oxidation with sulfate reduction, denitrification, or aerobic respiration to obtain energy for nitrogen fixation, which is in agreement with other studies of this class [96, 107, 108].

### Isolation of potential dominant diazotrophs from chemolithoautotrophic *Campylobacterota*

**Figure 5:**
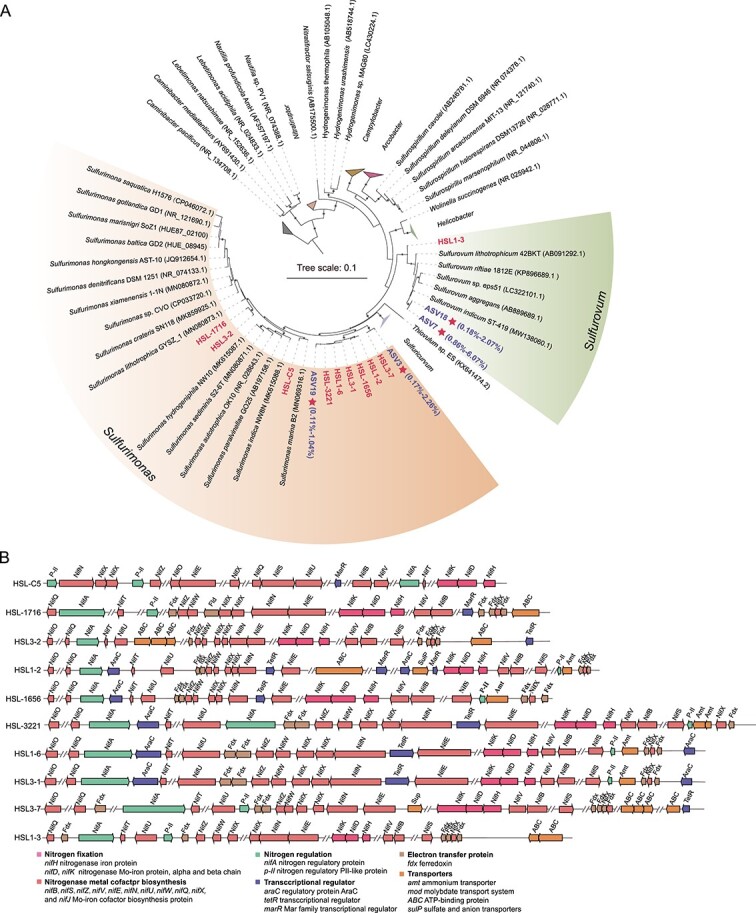
**Phylogenetic analysis and nitrogen fixation gene clusters of ten isolates from *Campylobacterota*.** A, Phylogenetic tree based on 16S rRNA gene sequences from the representative ASVs of the genera *Sulfurimonas* and *Sulfurovum* and the isolates in this study. Only ASVs representing >1% of the communities in at least one sample are shown. The scale bar represents 1.0 nucleotide replacements per site. B, Gene neighborhoods of *nifHDK* include nitrogenase metal cofactor biosynthesis genes, regulatory nitrogen fixation genes, transcriptional regulator genes, electron transfer genes and transporter genes.

Ten strains named HSL-C5, HSL1–2, HSL1–6, HSL3–1, HSL3–2, HSL3–7, HSL-3221, HSL-1716, HSL-1656, and HSL1–3 were successfully isolated from mangrove sediments (Fig. 5A). They shared the highest 16S rRNA gene sequence similarities with members from the genera *Sulfurimonas* and *Sulfurovum* of the phylum *Campylobacterota* (Table S9), which were two predominance genera in *in situ* mangrove sediments by 16S rRNA gene from metagenomics and amplicon sequencing (See online supplementary material for a color version of Figs S18 and S19). Furthermore, phylogenetic tree based on the 16S rRNA gene sequences of ASVs from the two genera and our isolates showed that strain HSL1–3 was most closely related to the two most predominant members represented by ASV7 (up to 6.07% relative abundance) and ASV18 (up to 2.07% abundance) from the genus *Sulfurovum*, with 97.14% and 97.03% sequence identity, respectively (Fig. 5A and Table S10). Strain HSL3–7 formed a cluster with the most dominant ASV3 (up to 2.46% abundance) from the genus *Sulfurimonas* with 99.03% sequence identity. Strain HSL-3221 corresponded to the second most predominant ASV19 (up to 1.04% abundance) of the genus *Sulfurimonas* with 98.51% sequence identity (Fig. 5A and Table S10). Thus, these isolates represented the predominant members of the genera *Sulfurimonas* and *Sulfurovum* in *in situ* mangrove sediments. Additionally, upon evaluating average AAI [109] and performing a genome-based phylogenomic analysis [110], these 10 strains were assigned to 5 genera including 3 potentially new genera in the family *Sulfurimonadaceae* (See online supplementary material for a color version of Figs S20 and S21).

All 10 isolates harbored a complete nitrogen fixation gene cluster encoding the nitrogenase designated NifHDKENB (Fig. 5B). Gene neighborhood analyses of these strains revealed that electron transfer proteins, regulatory proteins, and those necessary for nitrogenase cofactor biosynthesis were encoded among the *nif* gene clusters (Fig. 5B), which was also found in previous reports [15, 111]. Furthermore, physiological characterization revealed that all 10 isolates were obligate chemolithoautotrophs, and none of the organic compounds tested supported their growth as carbon and energy sources. Genes involved in the oxidation of sulfur and hydrogen and the reduction of various terminal acceptors in these 10 isolates are shown in Table S11, which could supply energy for nitrogen fixation. Indeed, nitrogen fixation is not common in chemolithoautotrophic *Campylobacterota* and so far only described for the member of *Sulfuricurvum kujiense* isolated from underground crude oil storage [112]. Thus, *Campylobacterota* strains containing nitrogenase genes identified herein may possess a competitive advantage in nitrogen-limited mangrove sediments. Compared with heterotrophic nitrogen-fixing bacteria [28, 113], fewer chemolithoautotrophic diazotrophs have been cultured by far. Prior to our study, only several bacteria belonging to the phyla *Pseudomonadota* and *Aquificota* have been isolated from freshwater, mine wastes, salt marshes, and hot springs, which could grow chemolithoautotrophicly using reduced sulfur compounds, hydrogen, As, or Sb as an energy source to fix N_2_ [19, 114–117]*.*

### Nitrogen fixation activity in the isolates and mangrove sediments

**Figure 6:**
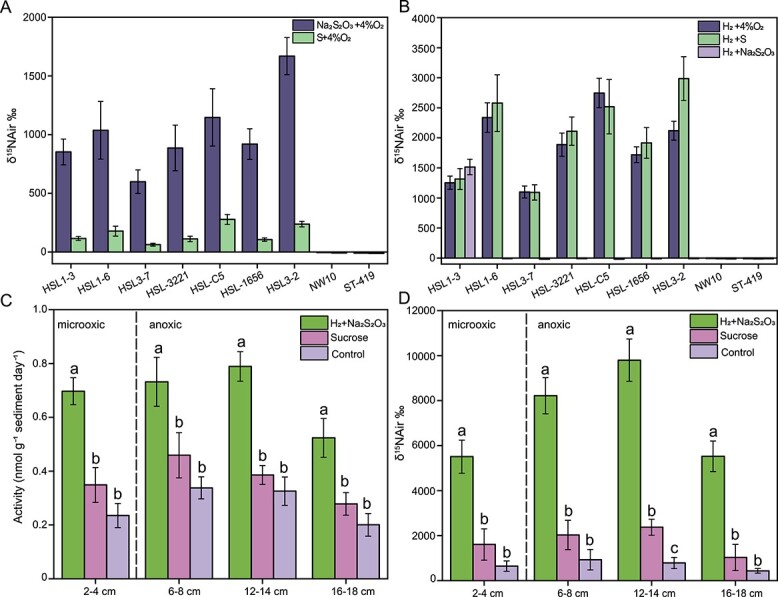
**Nitrogen fixation activities in *Campylobacterota* representative strains and mangrove sediments*.*** A, ^15^N abundance based on the ^15^N_2_ incorporation assay for seven *Campylobacterota* strains incubated with different reduced sulfur compounds as the energy source. B, ^15^N abundance based on the ^15^N_2_ incorporation assay for seven *Campylobacterota* strains incubated with H_2_ as the sole energy source coupled with different electron acceptors. The related type strains lacking the *nif* gene cluster are used as controls. C, Acetylene reduction assay of nitrogenase activity in four sediment layers incubated under chemolithoautotrophic and heterotrophic conditions. D, ^15^N abundance based on the ^15^N_2_ incorporation assay for four sediments incubated under chemolithoautotrophic and heterotrophic conditions. Standard deviations are indicated by error bars. The letters above the columns indicate statistically significant differences by Student’s *t*-test (*P* < 0.05).

The seven active strains that exhibited robust growth were selected to determine their ability to fix ^15^N_2_ intracellularly, using the ^15^N-labeled isotope analyses. The results showed that these seven strains were capable of fixing ^15^N_2_ with N_2_ as the sole nitrogen source under a gas mixture of ^15^N_2_:CO_2_ (Fig. 6A and B). As a comparison, strains *Sulfurimonas hydrogeniphila* NW10^T^ and *Sulfurovum indium* ST-419^T^, which lacked nitrogen fixation gene clusters, could not perform nitrogen fixation. As for the energy sources, hydrogen and sulfur compounds such as thiosulfate and elemental sulfur could be utilized with hydrogen as a preferred energy source, when these strains utilize oxygen, thiosulfate (only strain HSL1–3), or elemental sulfur as the sole electron acceptor (Fig. 6A and B). Nitrogen fixation is linked to hydrogen formation [118]. Hence, the recovery of energy via hydrogen oxidation could minimize the energy losses during nitrogen fixation. Combining N fixation with hydrogen oxidation to save energy has been described for *Cyanobacteriota* but has also been mentioned for other members of the phylum *Pseudomonadota* such as the genera *Thermochromatium* and *Rhodospirillum* [119, 120]. Furthermore, ^15^N_2_ fixation was completely inhibited by the addition of ammonia at both low and high concentrations (1 and 40 mM NH_4_Cl), and inhibited to some extent by the addition of 1 mM NaNO_3_ and NaNO_2_ in all seven strains (See online supplementary material for a color version of Fig. S22). In contrast, organic nitrogen compounds such as urea and amino acids did not inhibit their nitrogen fixation activity (See online supplementary material for a color version of Fig. S22), which is attributed to these isolates not being able to grow on these compounds as nitrogen sources. Additionally, these seven strains were able to fix ^15^N_2_ at both low and high oxygen concentrations (4% and 20% O_2_), which may be due to their antioxidant systems [121, 122].

To verify the contribution of chemolithoautotrophs to nitrogen fixation in mangrove sediments, we carried out activity measurements by acetylene reduction assay and ^15^N_2_ incorporation assay (Fig. 6 C and D). Based on the measured redox potential and the highest transcript expression detected in this study, four sediment layers (0–2, 6–8, 12–14, and 16–18 cm) were chosen to examine the nitrogen-fixing activity. The results showed that the autotrophic diazotrophic activities ranged from 0.52 ± 0.07 to 0.79 ± 0.06 nmol g^−1^ day^−1^, which were significantly higher than the heterotrophic diazotrophic activities (0.28 ± 0.04 to 0.46 ± 0.08 nmol g^−1^ day^−1^) in mangrove sediment samples under both microoxic and anoxic conditions (Fig. 6C). Without supplementation of any carbon or energy source, the nitrogenase activity of the original sediments was merely 0.19 ± 0.04 to 0.34 ± 0.04 nmol g^−1^ day^−1^ (Fig. 6C). In addition, the δ^15^N values were significantly higher in the cultures amended with inorganic carbon and energy sources than in the treatments with organics or in the original sediments after 10 days of incubation (Fig. 6D).

### Implications for chemolithoautotrophic diazotrophy in mangrove sediments

Taken together, our findings imply that chemolithoautotrophic diazotrophy rather than heterotrophic diazotrophy dominates in organic-rich and nitrogen-limited mangrove sediment habitats, which may be attributed to the low redox potential and abundant reduced inorganic compounds such as H_2_ and H_2_S in these setting. Generally, in marine sediments, when Eh < 0 mV, the sediment is strongly reductive, and when the Eh values range from 0 to 200 mV, the sediment is slightly reductive [123, 124]. In our study, sediments <4 cm exhibited highly reduced conditions with Eh < 0 mV (See online supplementary material for a color version of Fig. S3 J). The negative redox potential in deeper sediments except surface sediments indicates that the mangrove sediments are mostly water-logged without much periodical aeration, which is consistent with the microbiological data. Moreover, aerobic microorganisms consume oxygen during the decomposition of organic matter [125], which also leads to oxygen depletion with depth increase. However, considering the interference of mangrove root extension and the burrowing activity of polychaetes and crabs [126], the redox gradients in anoxic sediments may be dynamic. Besides the low redox potential, abundant reduced inorganic compounds such as H_2_ and H_2_S in these settings are also essential factors for nitrogen fixation by chemolithoautotrophs, and they are derived from the anaerobic fermentation of organic matter and sulfate reduction in sediments, respectively.

**Figure 7:**
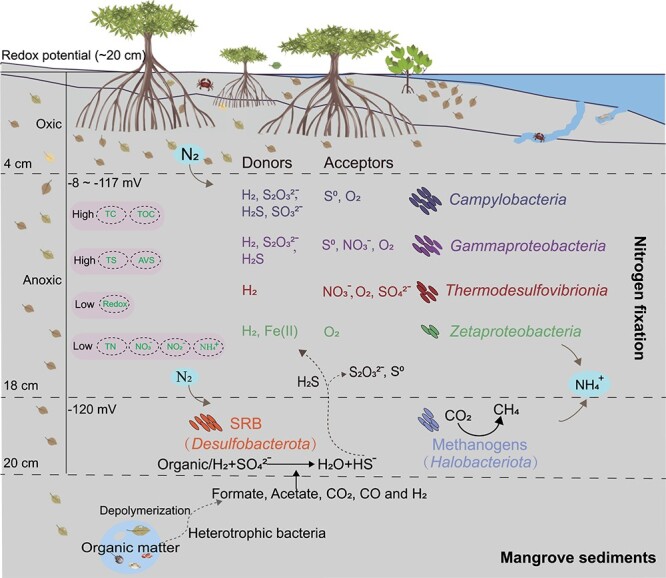
**Conceptual model of depth-related microbial nitrogen fixation in mangrove sediments.** Upper sediments (0–18 cm): The model highlights the significance of chemolithoautotrophy, a previously overlooked energy source driving dark nitrogen fixation. These chemolithoautotrophic diazotrophs utilize inorganic compounds such as hydrogen, reduced sulfur, and iron as energy sources, with oxygen, nitrate, and sulfur as terminal electron acceptors. H_2_ and H_2_S are produced by the degradation of organic matter and sulfate reduction, respectively. Deeper sediments (18–20 cm): The diazotrophs involved in dissimilatory sulfate reduction and methanogenesis are strongly enriched. Sulfate reduction is carried out utilizing the small molecule compounds produced from deeper sediments and resulting in abundant sulfide production.

Potential chemolithoautotrophic diazotrophs may fill an essential ecological niche, contributing to the initial accumulation of organic carbon and nitrogen in mangrove sediments and facilitating ecosystem productivity, which is similar to their roles in oligotrophic habitats such as tailings or glacier forefields [19, 20]. A conceptual model of depth and redox-related microbial nitrogen fixation by different energy sources was constructed (Fig. 7). In the upper 0–18 cm sediment layer (Eh = ~ −117 mV), chemolithoautotrophs are the most predominant and active diazotrophs, which utilize hydrogen, reduced sulfur species, and iron as the electron donors, with oxygen, nitrate, and sulfur as terminal electron acceptors. Moreover, carbon dioxide is used by these chemolithoautotrophs to form new organic carbon that feeds heterotrophs within the microbial assemblages. In the deeper sediments ~18–20 cm (Eh = −120 mV), diazotrophs involved in dissimilatory sulfate reduction and methanogenesis are strongly enriched. A large amount of sulfide is produced from sulfate-reducing bacteria via organic matter mineralization or H_2_ oxidation, and then diffuses upward, where it is further utilized by chemolithoautotrophic sulfur-oxidizing bacteria in upper sediments to form thiosulfate and even sulfate. Small molecule compounds such as formate, acetate, propionate, butyrate, H_2_, and CO_2_ originate from the anaerobic fermentation of macromolecules in deeper layers of mangrove sediments [36].

## Conclusions

The findings from this research significantly enhance our understanding of biological nitrogen fixation within coastal eutrophic sediments, shedding light on the ecological significance of chemolithoautotrophic organisms in nitrogen metabolism. Our results showed that an unexpectedly diverse assemblage of chemolithoautotrophs including *Campylobacterota*, *Pseudomonadota*, and *Nitrospirota* are the predominant and active nitrogen fixers in the surface sediments of mangroves. They play a pivotal role in carbon and sulfur elemental cycling by mitigating nitrogen shortages in mangrove sediments that are rich in carbon and sulfur. From a metabolic standpoint, reduced sulfur, hydrogen, and iron serve as the principal energy sources for microbial chemosynthesis. These chemolithoautotrophic diazotrophs are deemed crucial not only in mangrove sediments but also in other habitats where there is a disparity between carbon and nitrogen and a richness in reduced inorganic compounds. Future studies are needed to quantify the contribution of these chemolithoautotrophs to the total amount of nitrogen fixation *in situ*.

## Supplementary Material

Supplement_Data_wrae119

Supplement_Table_wrae119

## Data Availability

All metagenomic and metatranscriptomic raw reads used in this study are available in NCBI under accessions SAMN37418899–37418908 (BioProject PRJNA1017975) and SAMN37429165–37429174 (BioProject PRJNA1018229), respectively. The sequences from 16S rRNA gene and genome in 10 strains are available in NCBI with the accession numbers shown in Table S9. The assemblies, reference gene catalog, all MAGs, and phylogenetic trees could be found in figshare (https://doi.org/10.6084/m9.figshare.24331438).
