# Supplementary material for: Chemolithoautotrophic diazotrophs dominate dark nitrogen fixation in mangrove sediments
Source: ISME J. 2024 Jun 25;18(1):wrae119. doi: 10.1093/ismejo/wrae119 (PMC11474244; doi:10.1093/ismejo/wrae119)
Supplement: Supplement_Data_wrae119 [file supplement_data_wrae119.pdf]

Chemolithoautotrophic diazotrophs dominate dark nitrogen fixation in mangrove sediments

Shasha Wang[1#], Lijing Jiang[1#*], Zhuoming Zhao[1], Zhen Chen[1], Jun Wang[1], Karine Alain[3], Liang Cui[1], Yangsheng Zhong[1], Yongyi Peng[1], Qiliang Lai[1], Xiyang Dong[1,2*] and Zongze Shao[1,2*]

[1] Key Laboratory of Marine Genetic Resources, Third Institute of Oceanography, Ministry of Natural Resources of China; State Key Laboratory Breeding Base of Marine Genetic Resources; Fujian Key Laboratory of Marine Genetic Resources; Sino-French Laboratory of Deep-Sea Microbiology (MicrobSea), Xiamen 361005, PR China

[2] Southern Marine Science and Engineering Guangdong Laboratory (Zhuhai), Zhuhai 519000, PR China

[3] Univ Brest, CNRS, Ifremer, EMR6002 BIOMEX, Biologie Interactions et adaptations des Organismes en Milieu EXtrême, IRP 1211 MicrobSea, F-29280 Plouzané, France

# These authors contributed equally to this work.

* Corresponding authors: Zongze Shao, Key Laboratory of Marine Genetic Resources, Third Institute of Oceanography, Ministry of Natural Resources of China, 186 Daxue Road, Fujian 361005, China. Email: shaozz@163.com, Xiyang Dong, Key Laboratory of Marine Genetic Resources, Third Institute of Oceanography, Ministry of Natural Resources of China, 186 Daxue Road, Fujian 361005, China. Email: dongxiyang@tio.org.cn, and Lijing Jiang, Key Laboratory of Marine Genetic Resources, Third Institute of Oceanography, Ministry of Natural Resources of China, 186 Daxue Road, Fujian 361005, China. Email: jianglijing@tio.org.cn

Running title: Chemolithoautotrophic diazotrophs in mangrove sediments

Supplementary Figures

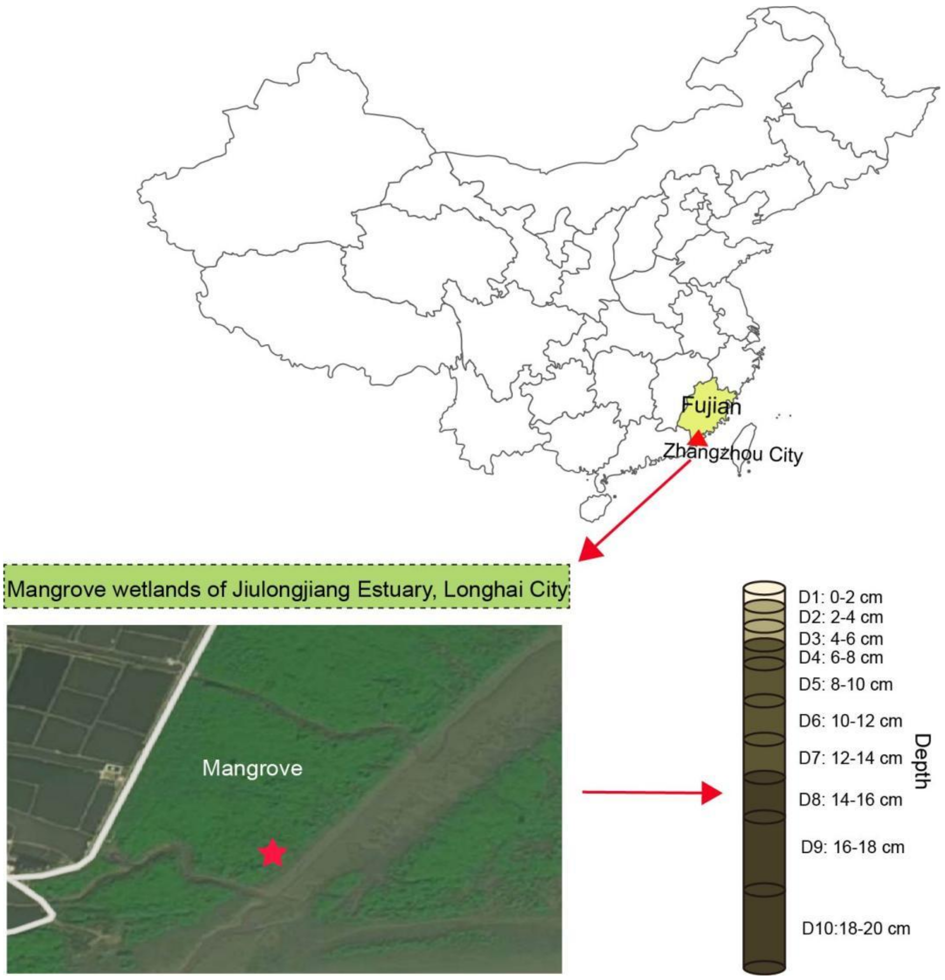

Fig. S1. Location of the sampling habitats at the Zhangzhou Mangrove wetland, China, which is dominated by *Kandelia obovata*. In this study site, one sediment core was collected using a custom-made sampler with a 20-cm depth and a 11-cm diameter. The sediment core was subdivided into 10-depths: 0-2, 2-4, 4-6, 6-8, 8-10, 10-12, 12-14, 14-16, 16-18, and 18-20 cm.

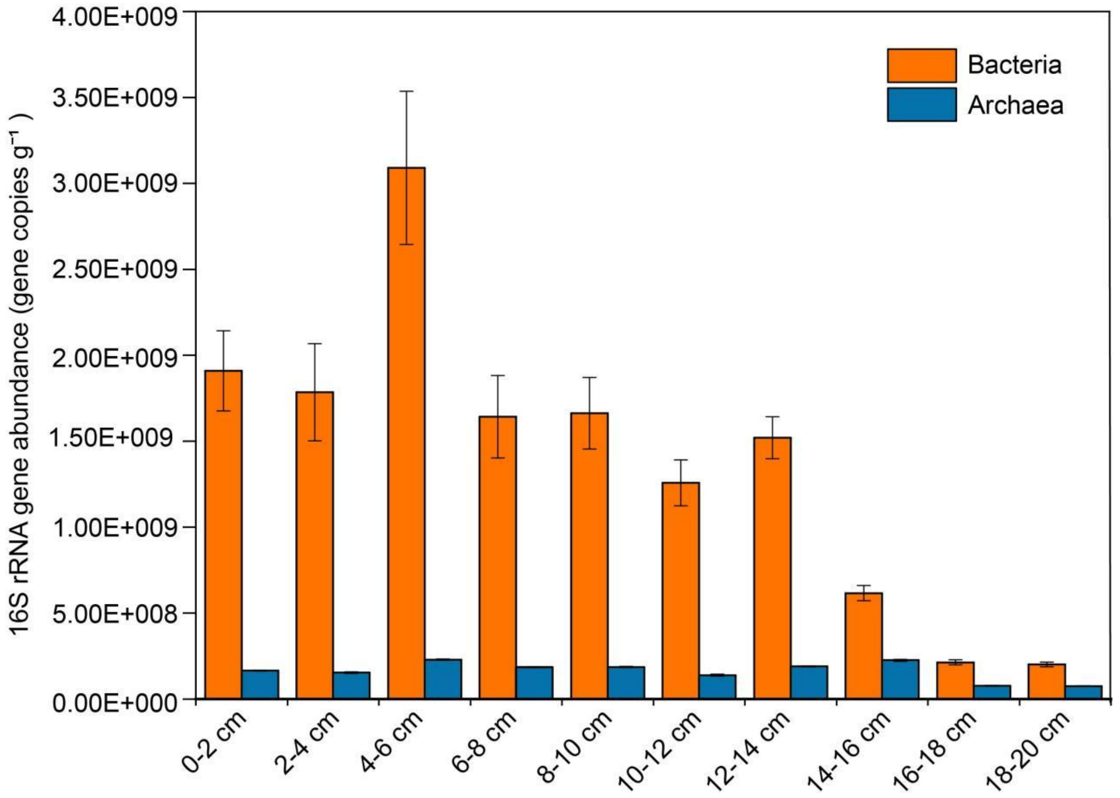

Fig. S2. Quantitative results of the abundance of bacterial and archaeal 16S rRNA gene from ten mangrove sediment samples. Standard deviations are indicated by error bars.

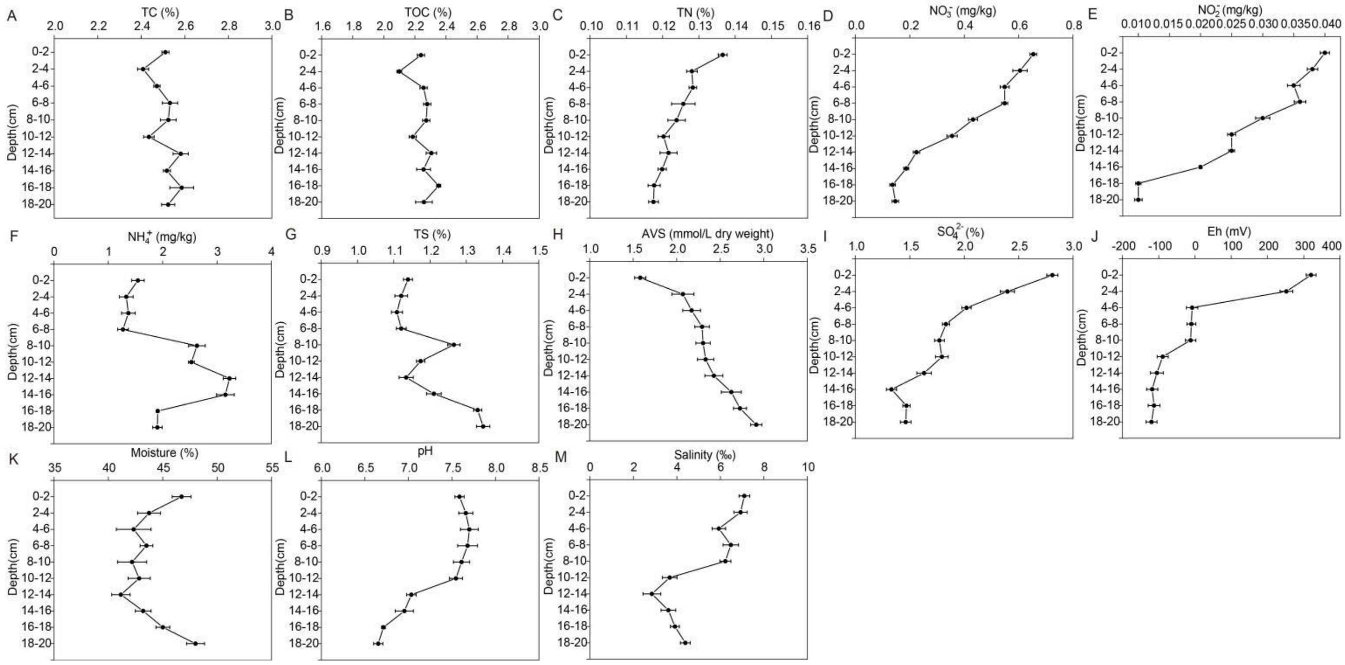

Fig. S3. Vertical distribution of various physicochemical characteristics across the depths of mangrove sediments. Data include the concentrations of TC (A), TOC (B), TN (C), NO_3^- (D), NO_2^- (E), NH_4^+ (F), TS (G), AVS (H), SO_4^{2-} (I), Eh (J), moisture (K), pH (L), and salinity (M). Error bars represent standard deviation.

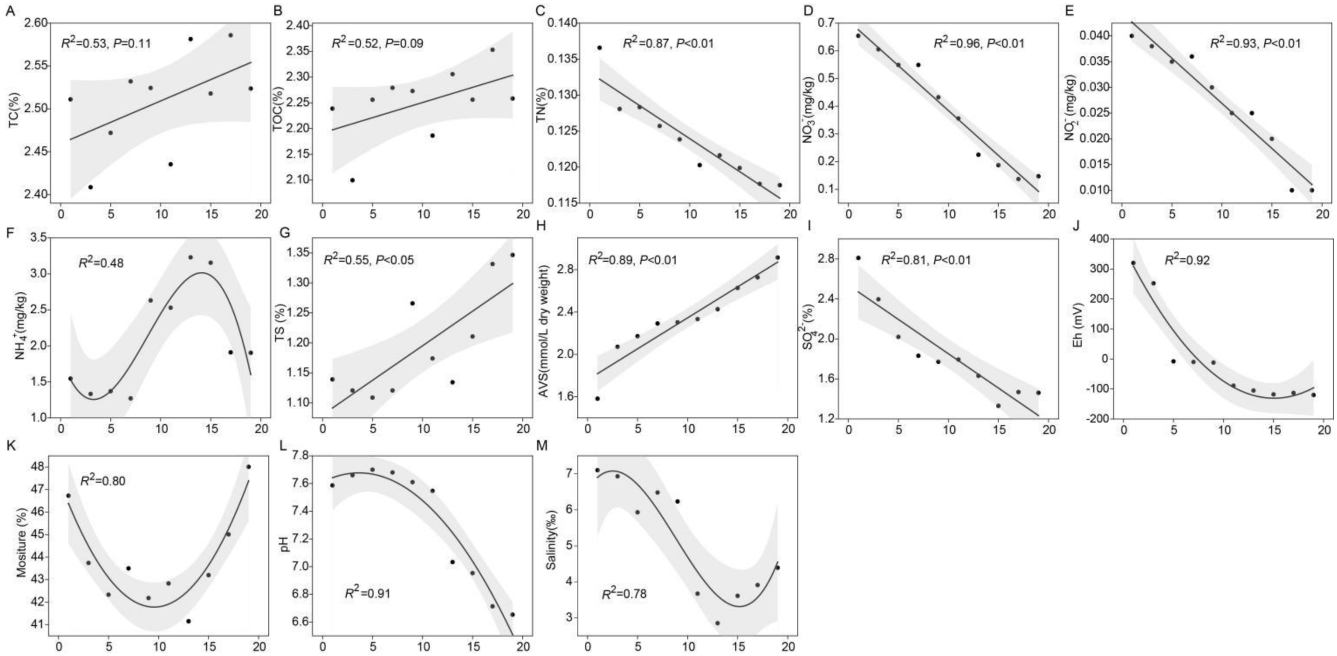

Fig. S4. Relationships between physicochemical properties and depth of mangrove sediments. Curve regression analysis of sediments properties TC (A), TOC (B), TN (C), NO_3^- (D), NO_2^- (E), NH_4^+ (F), TS (G), AVS (H), SO_4^{2-} (I), Eh (J), moisture (K), pH (L), and salinity (M) and depths. Black lines and gray shaded areas represent linear regressions and 95% confidence intervals, respectively. R^2 was obtained by linear regression analysis and P was obtained by Pearson's correlation analysis.

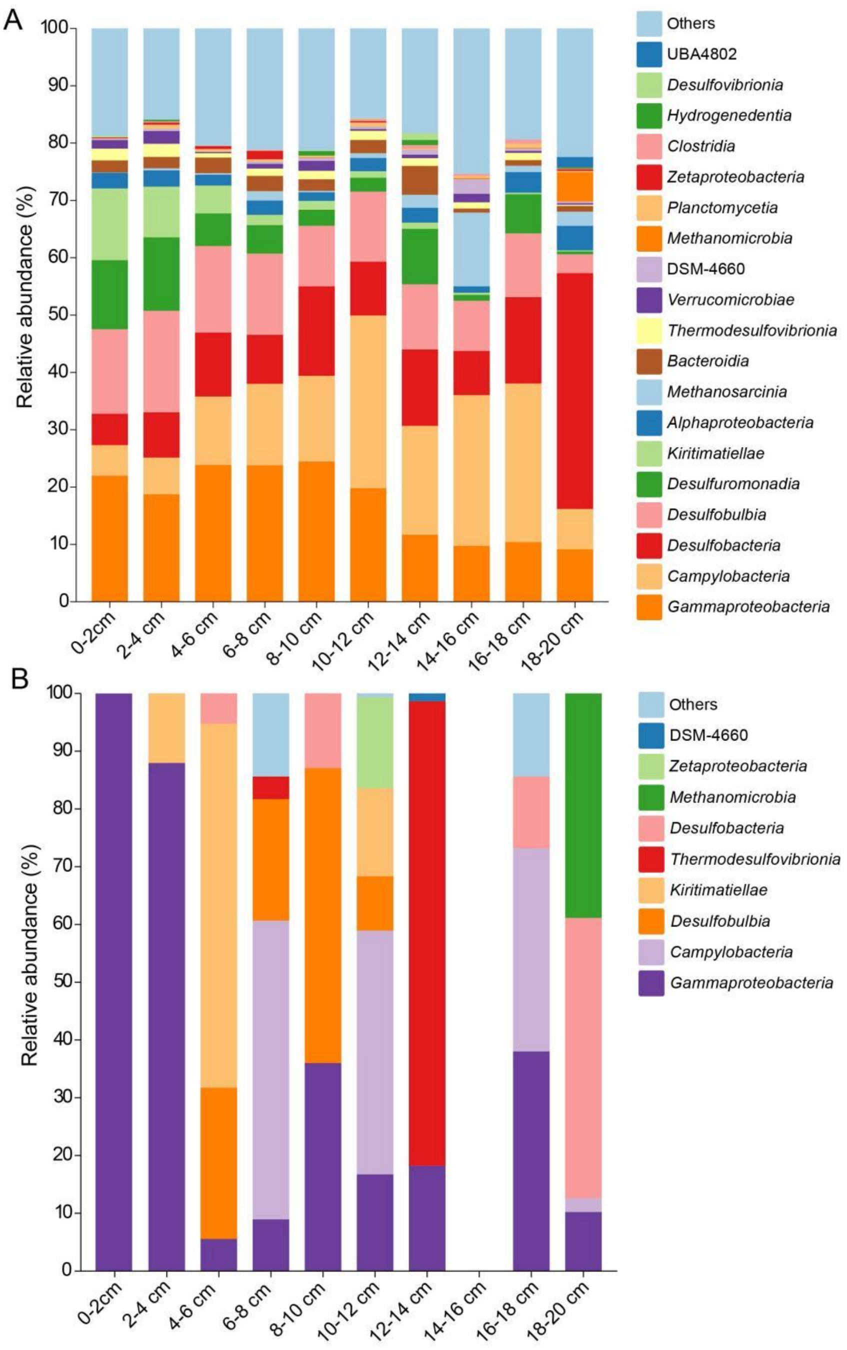

Fig. S5. Relative abundances of *nifH* genes at the class level at the metagenomics (A) and metatranscriptomics (B) from different mangrove sediment samples. Different taxonomic groups are represented by different colors. “Other” represents the unknown classification of the communities.

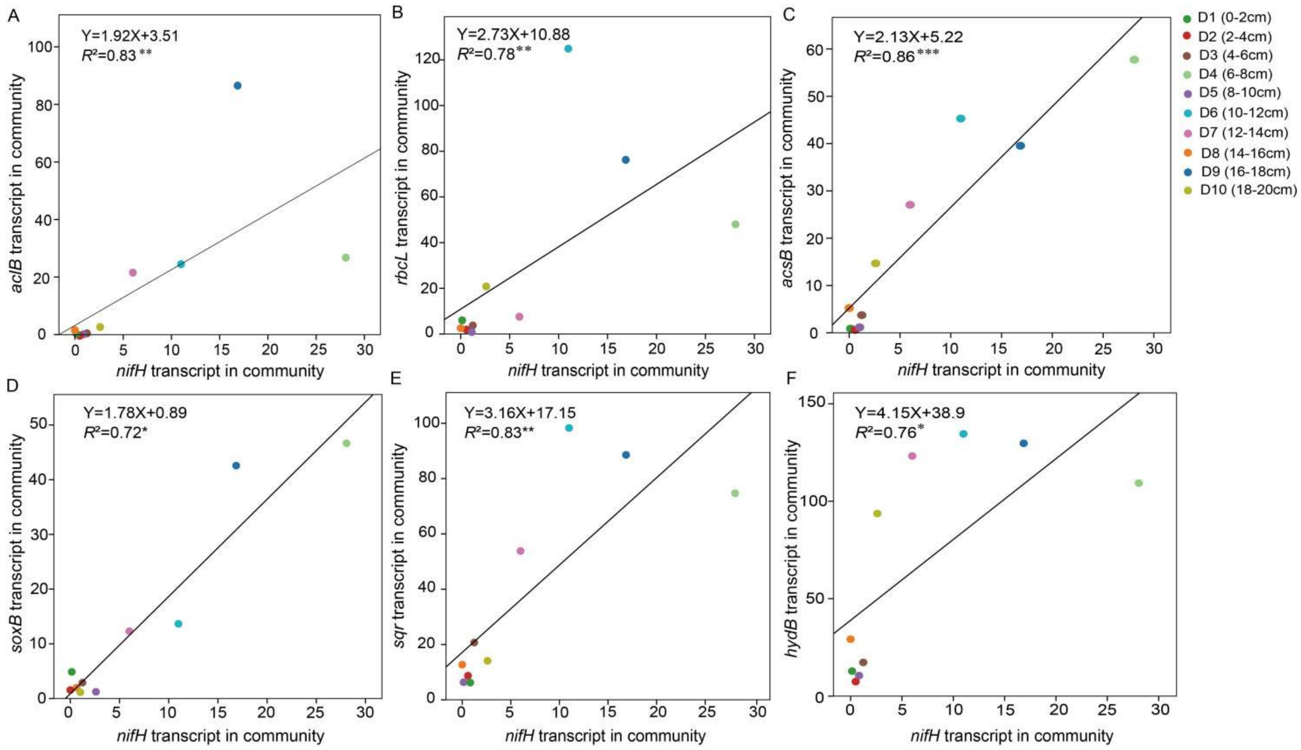

Fig. S6. Correlations between the transcriptional expression of *nifH* gene and other functional genes including *aclB*, *rbcL*, *acsB*, *sqr*, *soxB*, and *hydB* in mangrove sediments. Each point represents an individual sample. The solid line indicates the linear regression of the *nifH* gene and the functional genes. R^2 was obtained by linear regression analysis and significance levels are denoted with * ($0.01 < P < 0.05$), ** ($0.001 < P < 0.01$), and *** ($P < 0.001$).

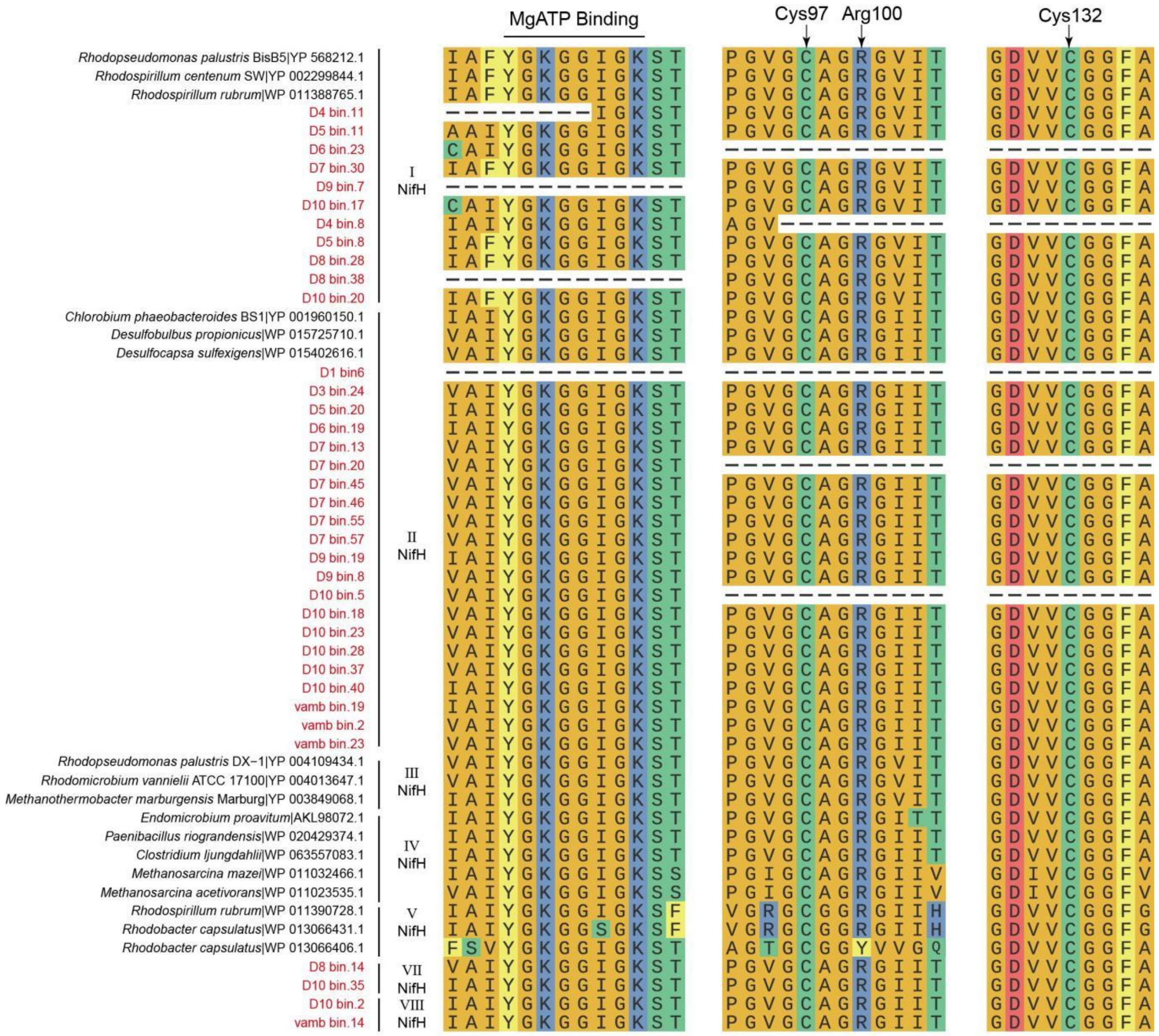

Fig. S7. NifH superfamily amino acid alignment. Multiple alignments of NifH superfamily sequences are shown in the region of active site residues responsible for MgATP binding and hydrolysis Fe_4-S_4 iron sulfur cluster binding.

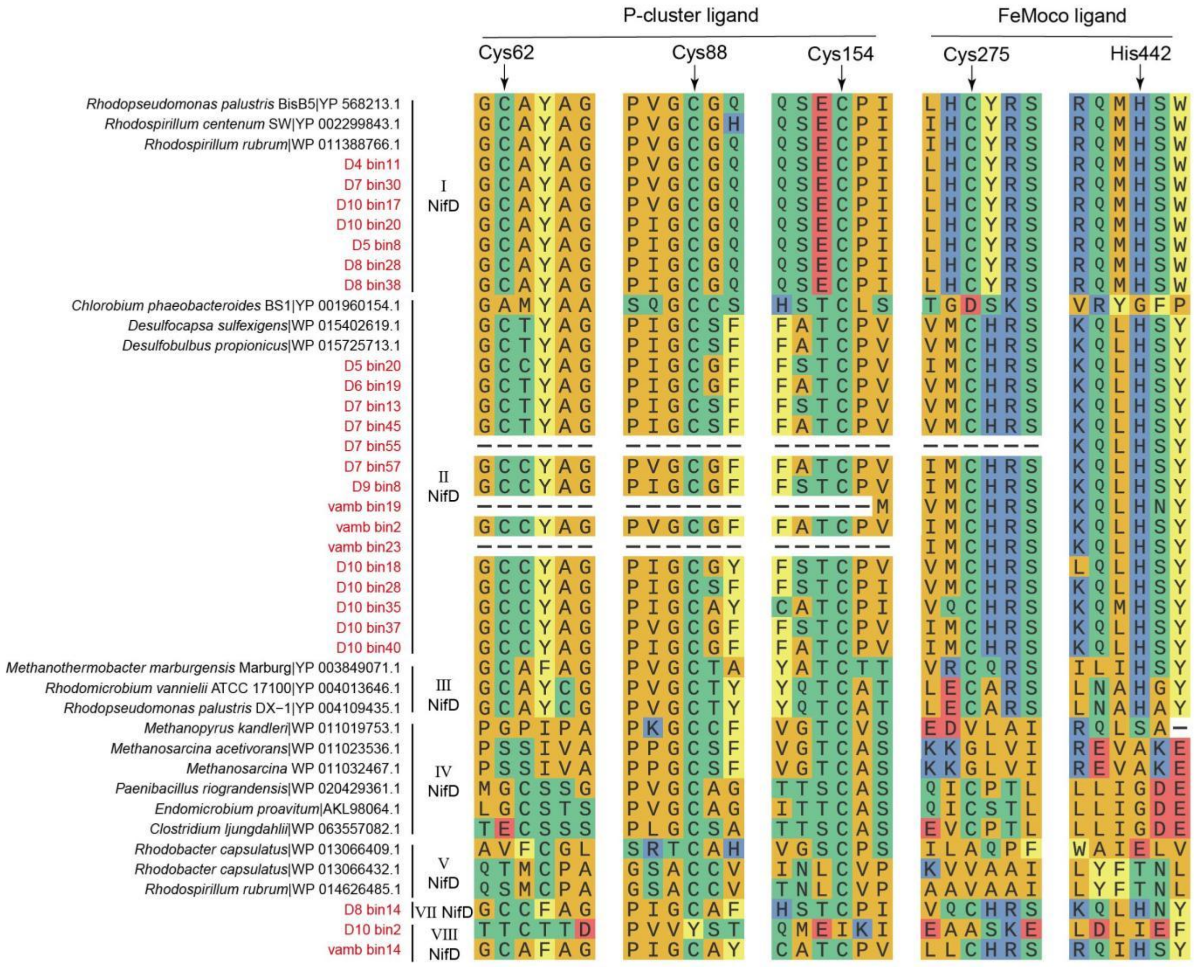

Fig. S8. NifD superfamily amino acid alignment. Multiple alignments of NifD superfamily sequences are shown in the region of active site residues responsible for coordination of the P-cluster and FeMo-cofactor within the molybdenum nitrogenase subunit NifD, and substrate bound to the FeMo-cofactor.

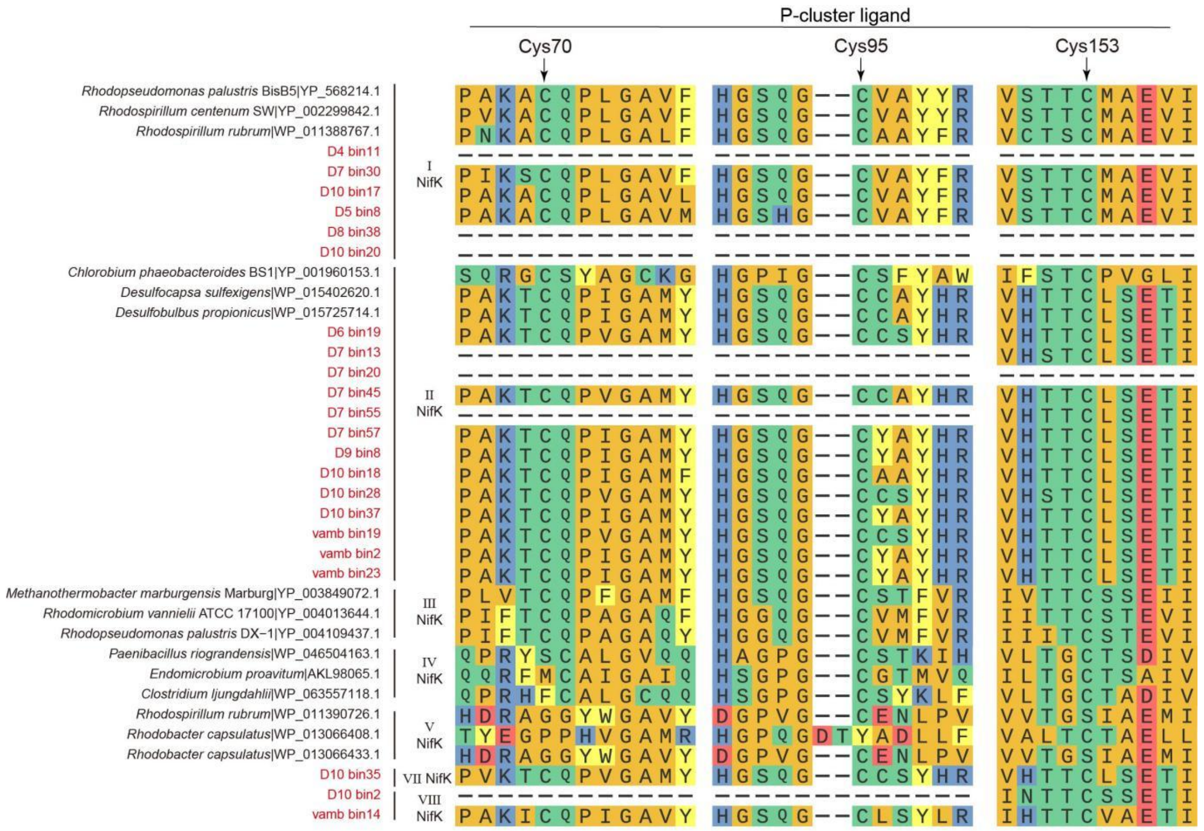

Fig. S9. NifK superfamily amino acid alignment. Multiple alignments of NifK superfamily sequences are shown in the region of active site residues responsible for coordination of the P-cluster within the molybdenum nitrogenase subunit NifK.

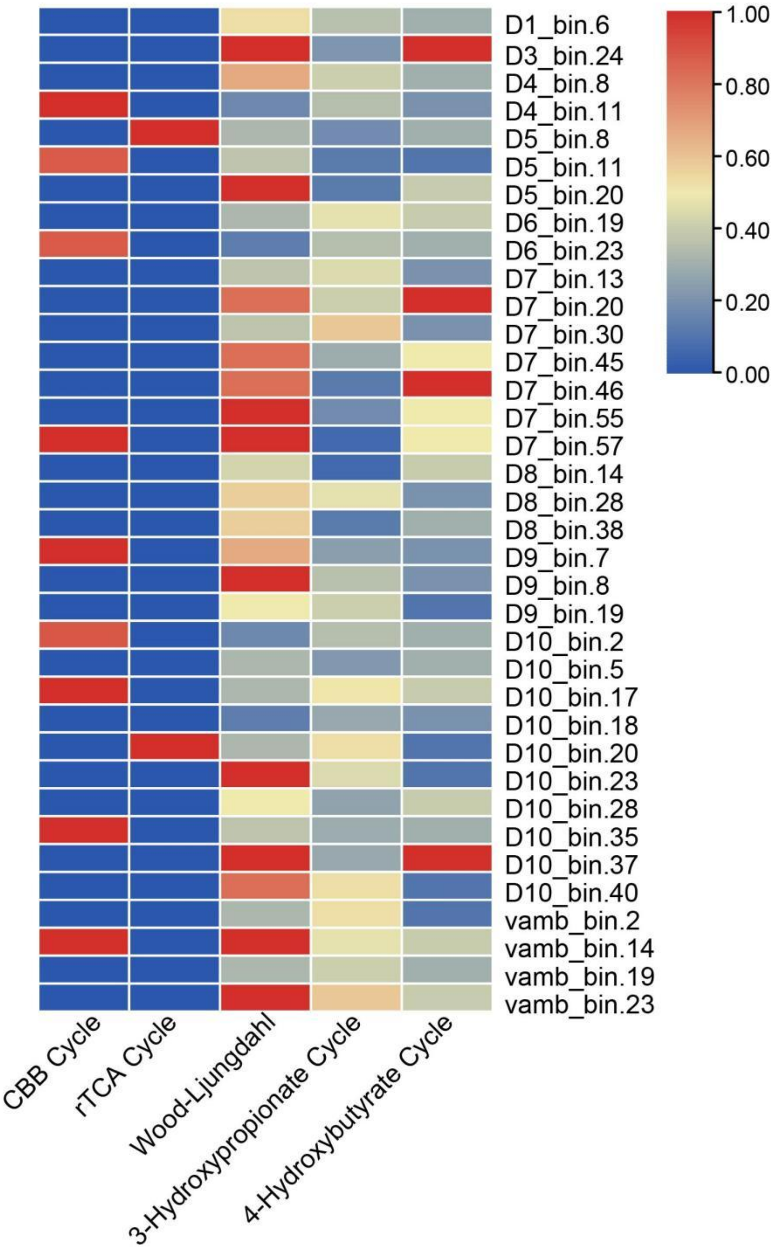

Fig. S10. The completeness of carbon fixation pathways by 36 putative nitrogen-fixating MAGs calculated by KEGG-Decoder.

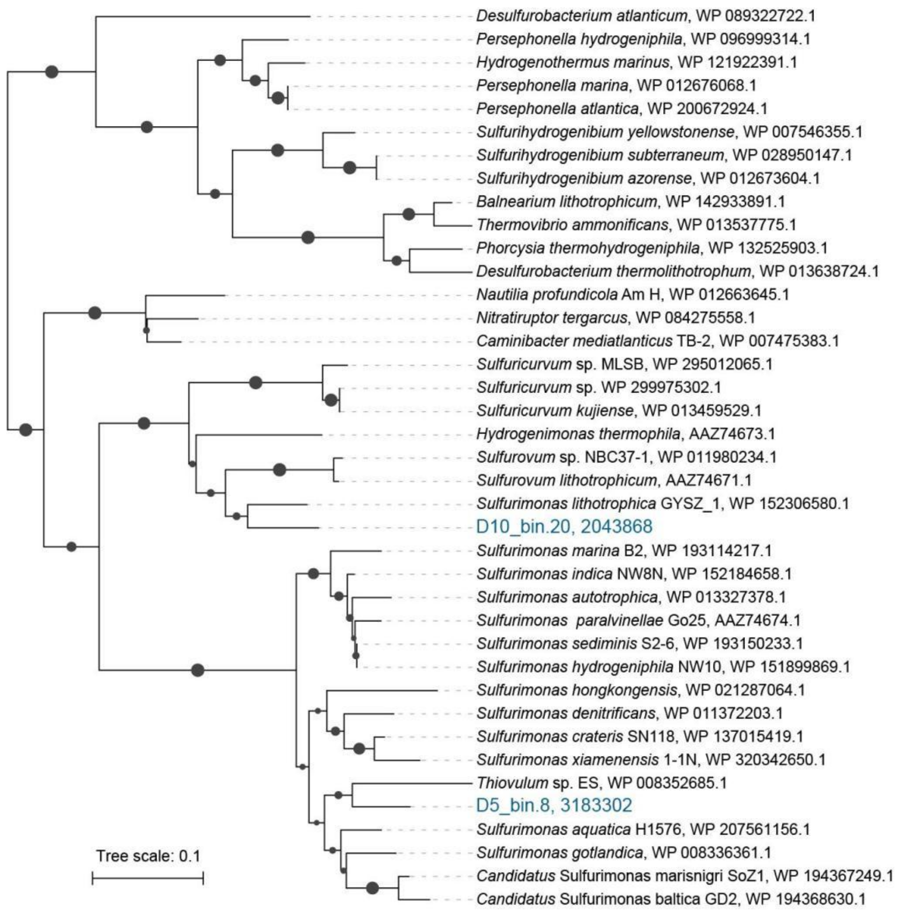

Fig. S11. Maximum-likelihood tree of amino acid sequences of AclB, a marker for carbon fixation. The tree shows sequences from mangrove sediments metagenome-assembled genomes obtained in this study (blue) alongside representative reference sequences (black). The subgroup of each reference sequence is denoted. Only bootstrap values ≥ 50% (based on 1000 bootstrap replicates) are shown at nodes as closed circles.

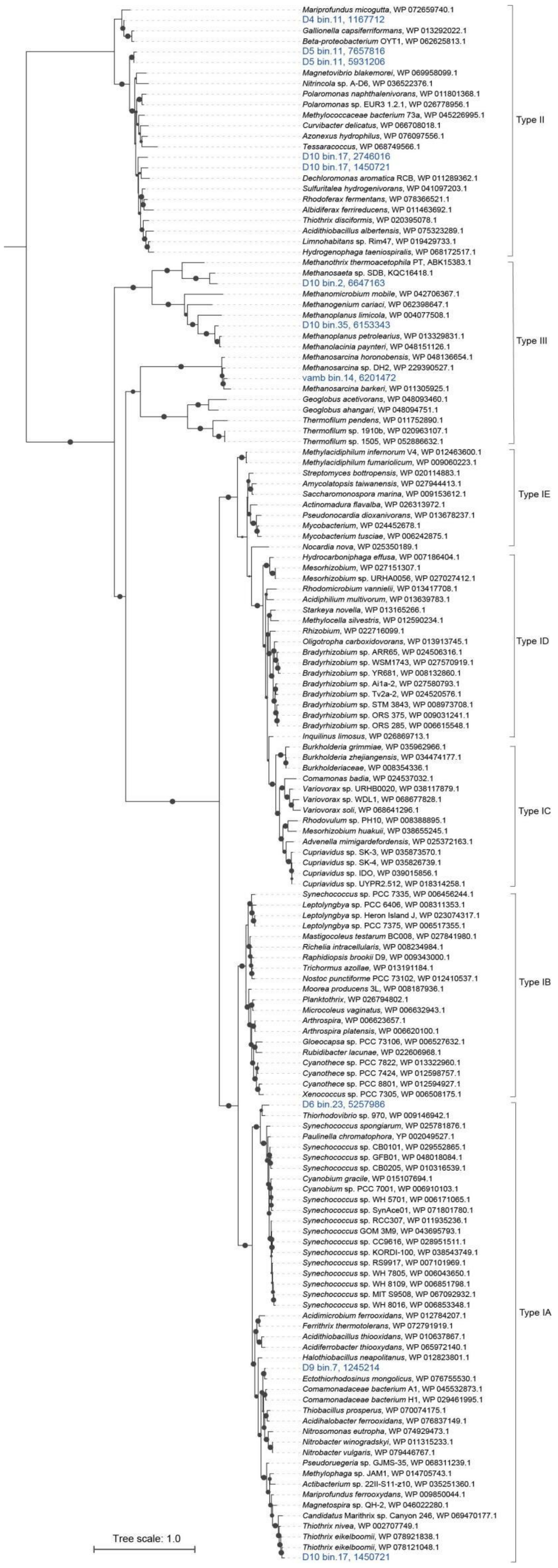
Mariprofundus micogutta, WP 072659740.1
D4 bin.11, 1167712
Gallionella capsiferriformans, WP 013292022.1
Beta-proteobacterium OYT1, WP 062625813.1
D5 bin.11, 7657816
D5 bin.11, 5931206
Magnetovibrio blakemorei, WP 069958099.1
Nitrincola sp. A-D6, WP 036522376.1
Polaromonas naphthalenivorans, WP 011801368.1
Polaromonas sp. EUR3 1.2.1, WP 026778956.1
Methylococcaceae bacterium 73a, WP 045226995.1
Curvibacter delicatus, WP 066708018.1
Azonexus hydrophilus, WP 076097556.1
Tessaracoccus, WP 068749566.1
D10 bin.17, 2746016
D10 bin.17, 1450721
Dechloromonas aromatica RCB, WP 011289362.1
Sulfuritalea hydrogenivorans, WP 041097203.1
Rhodoferax fermentans, WP 078366521.1
Albidiferax ferrireducens, WP 011463692.1
Thiothrix disciformis, WP 020395078.1
Acidithiobacillus albertensis, WP 075323289.1
Limnohabitans sp. Rim47, WP 019429733.1
Hydrogenophaga taeniospiralis, WP 068172517.1
Type II
Methanothrix thermoacetophila PT, ABK15383.1
Methanosaeta sp. SDB, KQC16418.1
D10 bin.2, 6647163
Methanomicrobium mobile, WP 042706367.1
Methanogenium cariaci, WP 062398647.1
Methanoplanus limicola, WP 004077508.1
D10 bin.35, 6153343
Methanoplanus petrolearius, WP 013329831.1
Methanolacinia paynteri, WP 048151126.1
Methanosarcina horonobensis, WP 048136654.1
Methanosarcina sp. DH2, WP 229390527.1
vamb bin.14, 6201472
Methanosarcina barkeri, WP 011305925.1
Geoglobus acetivorans, WP 048093460.1
Geoglobus ahangari, WP 048094751.1
Thermofilum pendens, WP 011752890.1
Thermofilum sp. 1910b, WP 020963107.1
Thermofilum sp. 1505, WP 052886632.1
Type III
Methylacidiphilum infernorum V4, WP 012463600.1
Methylacidiphilum fumariolicum, WP 009060223.1
Streptomyces bottropensis, WP 020114883.1
Amycolatopsis taiwanensis, WP 027944413.1
Saccharomonospora marina, WP 009153612.1
Actinomadura flavalba, WP 026313972.1
Pseudonocardia dioxanivorans, WP 013678237.1
Mycobacterium, WP 024452678.1
Mycobacterium tusciae, WP 006242875.1
Nocardia nova, WP 025350189.1
Type IE
Hydrocarboniphaga effusa, WP 007186404.1
Mesorhizobium, WP 027151307.1
Mesorhizobium sp. URHA0056, WP 027027412.1
Rhodomicrobium vannielii, WP 013417708.1
Acidiphilium multivorum, WP 013639783.1
Starkeya novella, WP 013165266.1
Methylocella silvestris, WP 012590234.1
Rhizobium, WP 022716099.1
Oligotropha carboxidovorans, WP 013913745.1
Bradyrhizobium sp. ARR65, WP 024506316.1
Bradyrhizobium sp. WSM1743, WP 027570919.1
Bradyrhizobium sp. YR681, WP 008132860.1
Bradyrhizobium sp. Ai1a-2, WP 027580793.1
Bradyrhizobium sp. Tv2a-2, WP 024520576.1
Bradyrhizobium sp. STM 3843, WP 008973708.1
Bradyrhizobium sp. ORS 375, WP 009031241.1
Bradyrhizobium sp. ORS 285, WP 006615548.1
Inquilinus limosus, WP 026869713.1
Type ID
Burkholderia grimmiae, WP 035962966.1
Burkholderia zhejiangensis, WP 034474177.1
Burkholderiaceae, WP 008354336.1
Comamonas badia, WP 024537032.1
Variovorax sp. URHB0020, WP 038117879.1
Variovorax sp. WDL1, WP 068677828.1
Variovorax soli, WP 068641296.1
Rhodovulum sp. PH10, WP 008388895.1
Mesorhizobium huakuii, WP 038655245.1
Advenella mimigardefordensis, WP 025372163.1
Cupriavidus sp. SK-3, WP 035873570.1
Cupriavidus sp. SK-4, WP 035826739.1
Cupriavidus sp. IDO, WP 039015856.1
Cupriavidus sp. UYPR2.512, WP 018314258.1
Type IC
Synechococcus sp. PCC 7335, WP 006456244.1
Leptolyngbya sp. PCC 6406, WP 008311353.1
Leptolyngbya sp. Heron Island J, WP 023074317.1
Leptolyngbya sp. PCC 7375, WP 006517355.1
Mastigocoleus testarum BC008, WP 027841980.1
Richelia intracellularis, WP 008234984.1
Raphidiopsis brookii D9, WP 009343000.1
Trichormus azollae, WP 013191184.1
Nostoc punctiforme PCC 73102, WP 012410537.1
Moorea producens 3L, WP 008187936.1
Planktothrix, WP 026794802.1
Microcoleus vaginatus, WP 006632943.1
Arthrospira, WP 006623657.1
Arthrospira platensis, WP 006620100.1
Gloeocapsa sp. PCC 73106, WP 006527632.1
Rubidibacter lacunae, WP 022606968.1
Cyanothece sp. PCC 7822, WP 013322960.1
Cyanothece sp. PCC 7424, WP 012598757.1
Cyanothece sp. PCC 8801, WP 012594927.1
Xenococcus sp. PCC 7305, WP 006508175.1
Type IB
D6 bin.23, 5257986
Thiorhodovibrio sp. 970, WP 009146942.1
Synechococcus spongiarum, WP 025781876.1
Paulinella chromatophora, YP 002049527.1
Synechococcus sp. CB0101, WP 029552865.1
Synechococcus sp. GFB01, WP 048018084.1
Synechococcus sp. CB0205, WP 010316539.1
Cyanobium gracile, WP 015107694.1
Cyanobium sp. PCC 7001, WP 006910103.1
Synechococcus sp. WH 5701, WP 006171065.1
Synechococcus sp. SynAce01, WP 071801780.1
Synechococcus sp. RCC307, WP 011935236.1
Synechococcus GOM 3M9, WP 043695793.1
Synechococcus sp. CC9616, WP 028951511.1
Synechococcus sp. KORDI-100, WP 038543749.1
Synechococcus sp. RS9917, WP 007101969.1
Synechococcus sp. WH 7805, WP 006043650.1
Synechococcus sp. WH 8109, WP 006851798.1
Synechococcus sp. MIT S9508, WP 067092932.1
Synechococcus sp. WH 8016, WP 006853348.1
Acidimicrobium ferrooxidans, WP 012784207.1
Ferrithrix thermotolerans, WP 072791919.1
Acidithiobacillus thiooxidans, WP 010637867.1
Acidiferrobacter thiooxydans, WP 065972140.1
Halothiobacillus neapolitanus, WP 012823801.1
D9 bin.7, 1245214
Ectothiorhodosinus mongolicus, WP 076755530.1
Comamonadaceae bacterium A1, WP 045532873.1
Comamonadaceae bacterium H1, WP 029461995.1
Thiobacillus prosperus, WP 070074175.1
Acidihalobacter ferrooxidans, WP 076837149.1
Nitrosomonas eutropha, WP 074929473.1
Nitrobacter winogradskyi, WP 011315233.1
Nitrobacter vulgaris, WP 079446767.1
Pseudoruegeria sp. GJMS-35, WP 068311239.1
Methylophaga sp. JAM1, WP 014705743.1
Actibacterium sp. 22II-S11-z10, WP 035251360.1
Mariprofundus ferrooxydans, WP 009850044.1
Magnetospira sp. QH-2, WP 046022280.1
Candidatus Marithrix sp. Canyon 246, WP 069470177.1
Thiothrix nivea, WP 002707749.1
Thiothrix eikelboomii, WP 078921838.1
Thiothrix eikelboomii, WP 078121048.1
D10 bin.17, 1450721
Type IA
Tree scale: 1.0

Fig. S12. Maximum-likelihood tree of amino acid sequences of RbcL, a marker for carbon fixation. The tree shows sequences from mangrove sediments metagenome-assembled genomes obtained in this study (blue) alongside representative reference sequences (black). Only bootstrap values ≥ 50% (based on 1000 bootstrap replicates) are shown at nodes as closed circles.

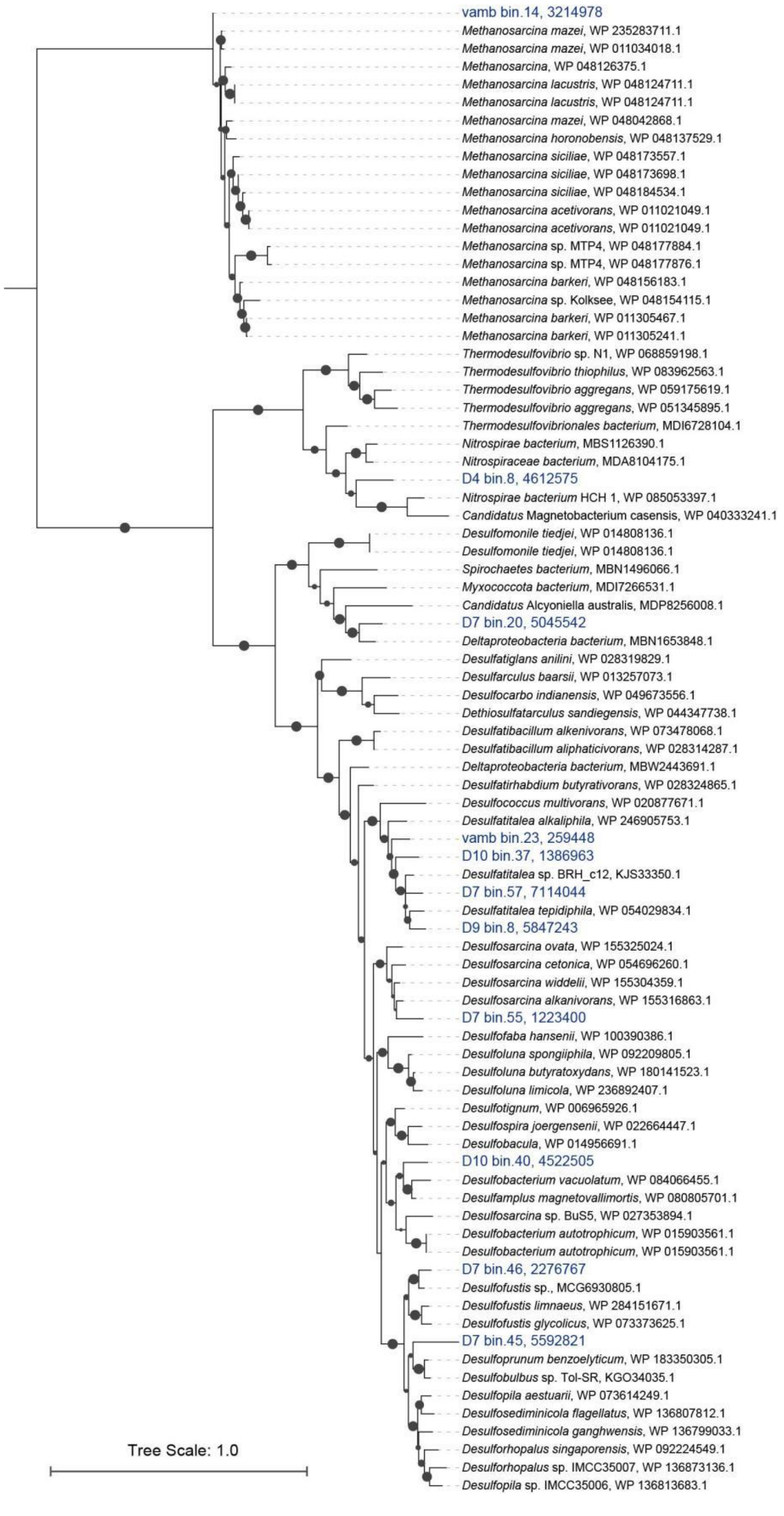

Fig. S13. Maximum-likelihood tree of amino acid sequences of AcsB, a marker for carbon fixation. The tree shows sequences from mangrove sediments metagenome-assembled genomes obtained in this study (blue) alongside representative reference sequences (black). Only bootstrap values ≥ 50% (based on 1000 bootstrap replicates) are shown at nodes as closed circles.

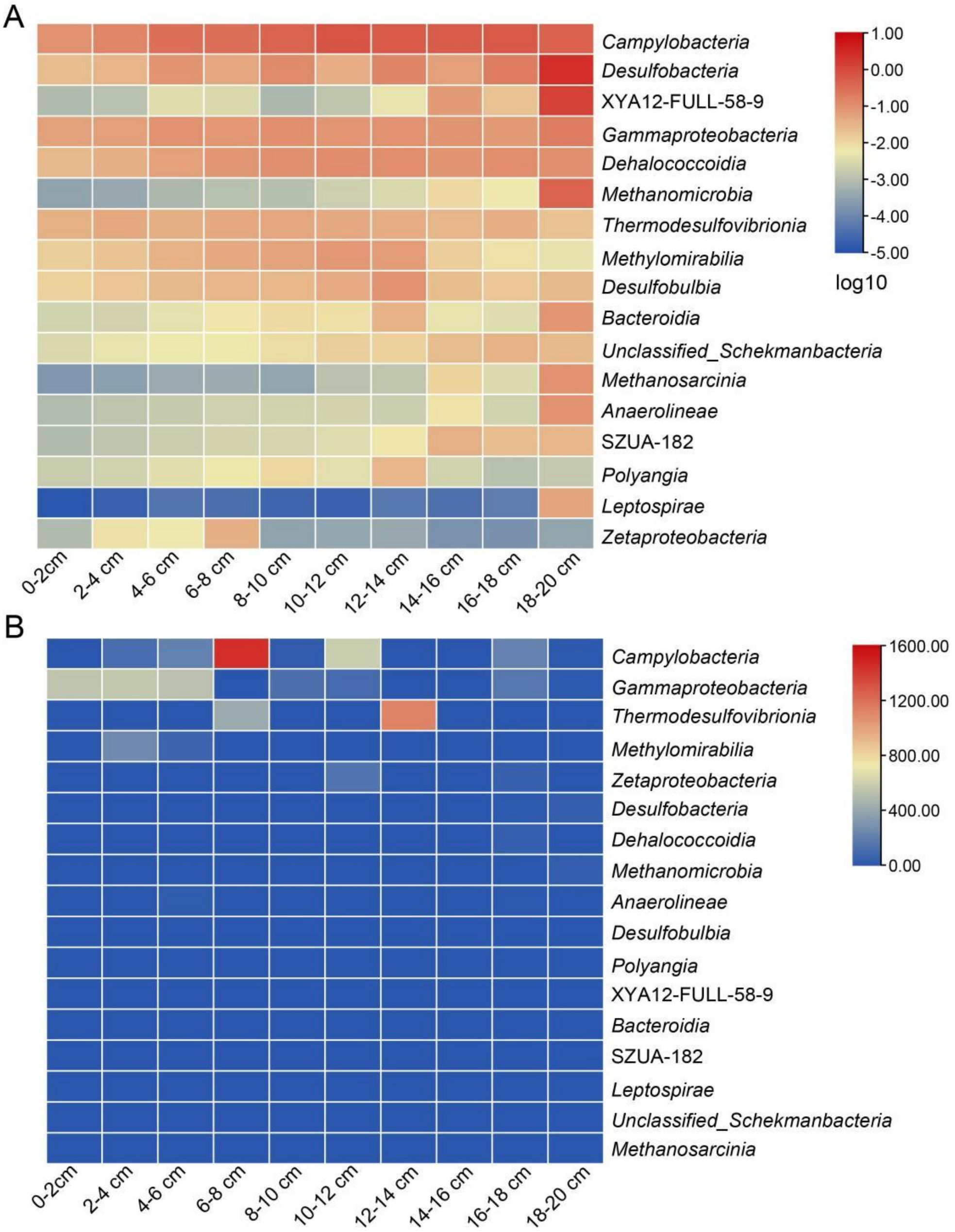

Fig. S14. Relative abundance (A) and transcript expression (B) of *nifH*-carrying MAGs at the class level in different mangrove sediment samples. Different taxonomic groups are represented by different colors.

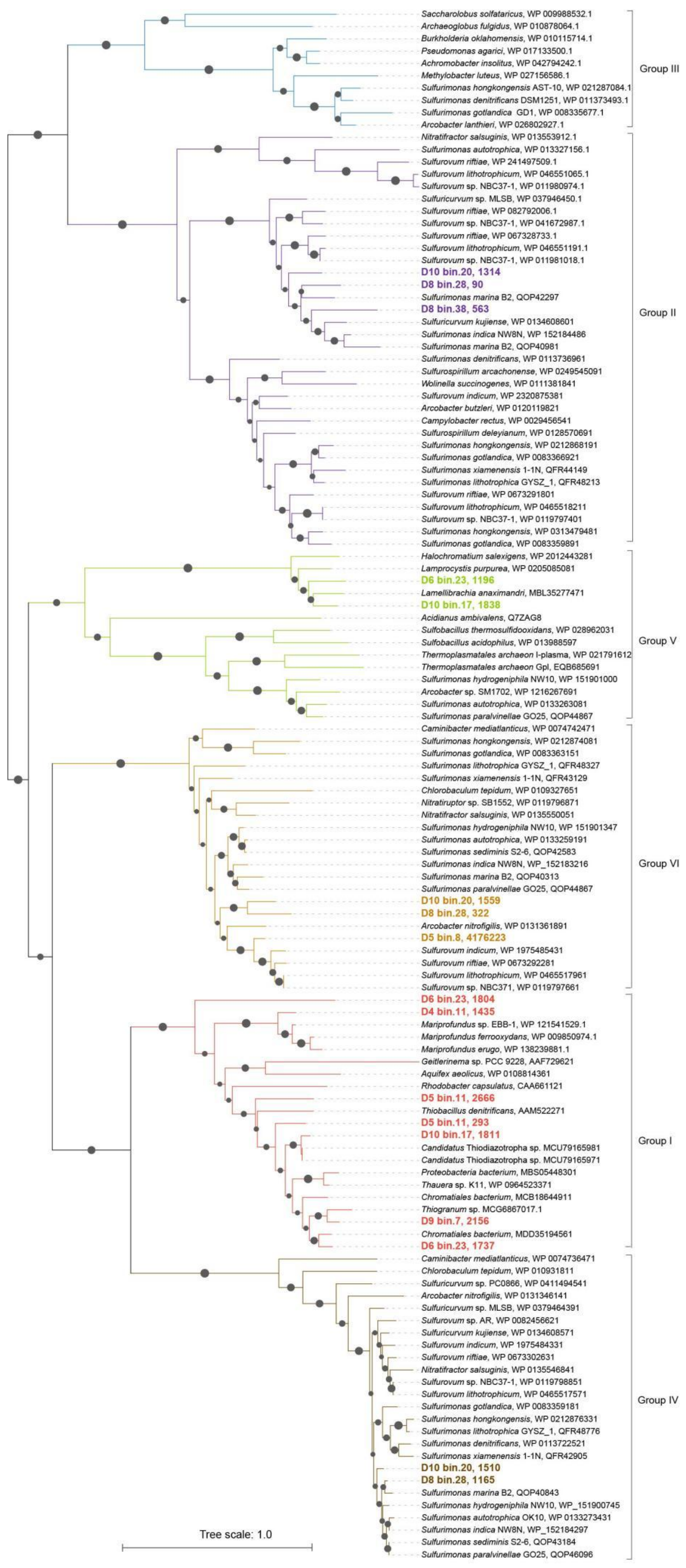
Saccharolobus solfataricus, WP 009988532.1
Archaeoglobus fulgidus, WP 010878064.1
Burkholderia oklahomensis, WP 010115714.1
Pseudomonas agarici, WP 017133500.1
Achromobacter insolitus, WP 042794242.1
Methylobacter luteus, WP 027156586.1
Sulfurimonas hongkongensis AST-10, WP 021287084.1
Sulfurimonas denitrificans DSM1251, WP 011373493.1
Sulfurimonas gotlandica GD1, WP 008335677.1
Arcobacter lanthieri, WP 026802927.1
Group III
Nitratifractor salsuginis, WP 013553912.1
Sulfurimonas autotrophica, WP 013327156.1
Sulfurovum riftiae, WP 241497509.1
Sulfurovum lithotrophicum, WP 046551065.1
Sulfurovum sp. NBC37-1, WP 011980974.1
Sulfuricurvum sp. MLSB, WP 037946450.1
Sulfurovum riftiae, WP 082792006.1
Sulfurovum sp. NBC37-1, WP 041672987.1
Sulfurovum riftiae, WP 067328733.1
Sulfurovum lithotrophicum, WP 046551191.1
Sulfurovum sp. NBC37-1, WP 011981018.1
D10 bin.20, 1314
D8 bin.28, 90
Sulfurimonas marina B2, QOP42297
D8 bin.38, 563
Sulfuricurvum kujiense, WP 0134608601
Sulfurimonas indica NW8N, WP 152184486
Sulfurimonas marina B2, QOP40981
Sulfurimonas denitrificans, WP 0113736961
Sulfurospirillum arcachonense, WP 0249545091
Wolinella succinogenes, WP 0111381841
Sulfurovum indicum, WP 2320875381
Arcobacter butzleri, WP 0120119821
Campylobacter rectus, WP 0029456541
Sulfurospirillum deleyianum, WP 0128570691
Sulfurimonas hongkongensis, WP 0212868191
Sulfurimonas gotlandica, WP 0083366921
Sulfurimonas xiamenensis 1-1N, QFR44149
Sulfurimonas lithotrophica GYSZ_1, QFR48213
Sulfurovum riftiae, WP 0673291801
Sulfurovum lithotrophicum, WP 0465518211
Sulfurovum sp. NBC37-1, WP 0119797401
Sulfurimonas hongkongensis, WP 0313479481
Sulfurimonas gotlandica, WP 0083359891
Group II
Halochromatium salexigens, WP 2012443281
Lamprocystis purpurea, WP 0205085081
D6 bin.23, 1196
Lamellibrachia anaximandri, MBL35277471
D10 bin.17, 1838
Acidianus ambivalens, Q7ZAG8
Sulfobacillus thermosulfidooxidans, WP 028962031
Sulfobacillus acidophilus, WP 013988597
Thermoplasmatales archaeon I-plasma, WP 021791612
Thermoplasmatales archaeon Gpl, EQB685691
Sulfurimonas hydrogeniphila NW10, WP 151901000
Arcobacter sp. SM1702, WP 1216267691
Sulfurimonas autotrophica, WP 0133263081
Sulfurimonas paralvinellae GO25, QOP44867
Group V
Caminibacter mediatlanticus, WP 0074742471
Sulfurimonas hongkongensis, WP 0212874081
Sulfurimonas gotlandica, WP 0083363151
Sulfurimonas lithotrophica GYSZ_1, QFR48327
Sulfurimonas xiamenensis 1-1N, QFR43129
Chlorobaculum tepidum, WP 0109327651
Nitratiruptor sp. SB1552, WP 0119796871
Nitratifractor salsuginis, WP 0135550051
Sulfurimonas hydrogeniphila NW10, WP 151901347
Sulfurimonas autotrophica, WP 0133259191
Sulfurimonas sediminis S2-6, QOP42583
Sulfurimonas indica NW8N, WP_152183216
Sulfurimonas marina B2, QOP40313
Sulfurimonas paralvinellae GO25, QOP44867
D10 bin.20, 1559
D8 bin.28, 322
Arcobacter nitrofigilis, WP 0131361891
D5 bin.8, 4176223
Sulfurovum indicum, WP 1975485431
Sulfurovum riftiae, WP 0673292281
Sulfurovum lithotrophicum, WP 0465517961
Sulfurovum sp. NBC371, WP 0119797661
Group VI
D6 bin.23, 1804
D4 bin.11, 1435
Mariprofundus sp. EBB-1, WP 121541529.1
Mariprofundus ferrooxydans, WP 009850974.1
Mariprofundus erugo, WP 138239881.1
Geitlerinema sp. PCC 9228, AAF729621
Aquifex aeolicus, WP 0108814361
Rhodobacter capsulatus, CAA661121
D5 bin.11, 2666
Thiobacillus denitrificans, AAM522271
D5 bin.11, 293
D10 bin.17, 1811
Candidatus Thiodiazotropha sp. MCU79165981
Candidatus Thiodiazotropha sp. MCU79165971
Proteobacteria bacterium, MBS05448301
Thauera sp. K11, WP 0964523371
Chromatiales bacterium, MCB18644911
Thiogranum sp. MCG6867017.1
D9 bin.7, 2156
Chromatiales bacterium, MDD35194561
D6 bin.23, 1737
Group I
Caminibacter mediatlanticus, WP 0074736471
Chlorobaculum tepidum, WP 010931811
Sulfuricurvum sp. PC0866, WP 0411494541
Arcobacter nitrofigilis, WP 0131346141
Sulfuricurvum sp. MLSB, WP 0379464391
Sulfurovum sp. AR, WP 0082456621
Sulfuricurvum kujiense, WP 0134608571
Sulfurovum indicum, WP 1975484331
Sulfurovum riftiae, WP 0673302631
Nitratifractor salsuginis, WP 0135546841
Sulfurovum sp. NBC37-1, WP 0119798851
Sulfurovum lithotrophicum, WP 0465517571
Sulfurimonas gotlandica, WP 0083359181
Sulfurimonas hongkongensis, WP 0212876331
Sulfurimonas lithotrophica GYSZ_1, QFR48776
Sulfurimonas denitrificans, WP 0113722521
Sulfurimonas xiamenensis 1-1N, QFR42905
D10 bin.20, 1510
D8 bin.28, 1165
Sulfurimonas marina B2, QOP40843
Sulfurimonas hydrogeniphila NW10, WP_151900745
Sulfurimonas autotrophica OK10, WP 0133273431
Sulfurimonas indica NW8N, WP_152184297
Sulfurimonas sediminis S2-6, QOP43184
Sulfurimonas paralvinellae GO25, QOP46096
Group IV
Tree scale: 1.0

Fig. S15. Maximum-likelihood tree of amino acid sequences of Sqr, a marker for sulfide oxidation. The tree shows sequences from mangrove sediments metagenome-assembled genomes obtained in this study (different colors for group I-VI) alongside representative reference sequences (black). Only bootstrap values ≥ 50% (based on 1000 bootstrap replicates) are shown at nodes as closed circles.

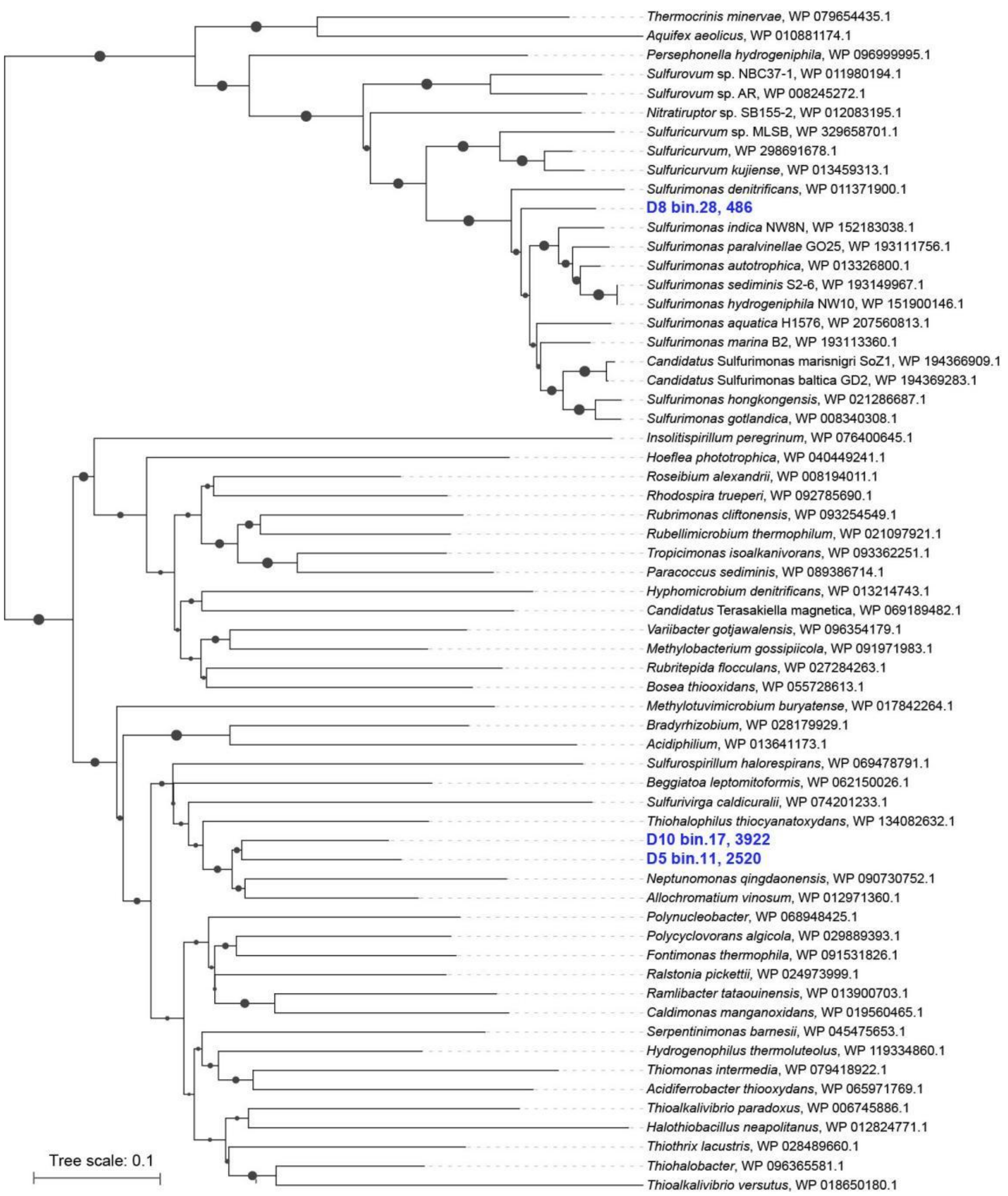

Fig. S16. Maximum-likelihood tree of amino acid sequences of SoxB, a marker for thiosulfate oxidation. The tree shows sequences from mangrove sediments metagenome-assembled genomes obtained in this study (blue) alongside representative reference sequences (black). Only bootstrap values ≥ 50% (based on 1000 bootstrap replicates) are shown at nodes as closed circles.

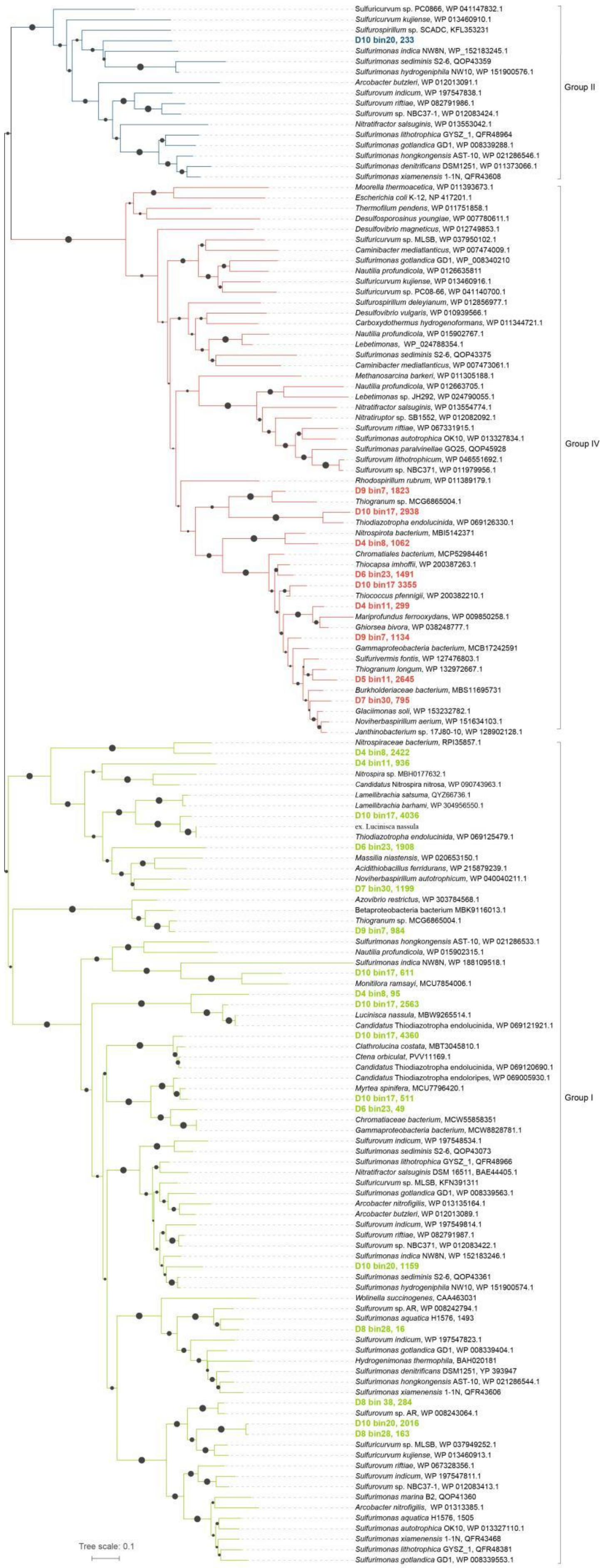

Sulfuricurvum sp. PC0866, WP 041147832.1
Sulfuricurvum kujiense, WP 013460910.1
Sulfurospirillum sp. SCADC, KFL353231
D10 bin20, 233
Sulfurimonas indica NW8N, WP_152183245.1
Sulfurimonas sediminis S2-6, QOP43359
Sulfurimonas hydrogeniphila NW10, WP 151900576.1
Arcobacter butzleri, WP 012013091.1
Sulfurovum indicum, WP 197547838.1
Sulfurovum riftiae, WP 082791986.1
Sulfurovum sp. NBC37-1, WP 012083424.1
Nitratifractor salsuginis, WP 013553042.1
Sulfurimonas lithotrophica GYSZ_1, QFR48964
Sulfurimonas gotlandica GD1, WP 008339288.1
Sulfurimonas hongkongensis AST-10, WP 021286546.1
Sulfurimonas denitrificans DSM1251, WP 011373066.1
Sulfurimonas xiamenensis 1-1N, QFR43608
Group II
Moorella thermoacetica, WP 011393673.1
Escherichia coli K-12, NP 417201.1
Thermofilum pendens, WP 011751858.1
Desulfosporosinus youngiae, WP 007780611.1
Desulfovibrio magneticus, WP 012749853.1
Sulfuricurvum sp. MLSB, WP 037950102.1
Caminibacter mediatlanticus, WP 007474009.1
Sulfurimonas gotlandica GD1, WP_008340210
Nautilia profundicola, WP 0126635811
Sulfuricurvum kujiense, WP 013460916.1
Sulfuricurvum sp. PC08-66, WP 041140700.1
Sulfurospirillum deleyianum, WP 012856977.1
Desulfovibrio vulgaris, WP 010939566.1
Carboxydothermus hydrogenoformans, WP 011344721.1
Nautilia profundicola, WP 015902767.1
Lebetimonas, WP_024788354.1
Sulfurimonas sediminis S2-6, QOP43375
Caminibacter mediatlanticus, WP 007473061.1
Methanosarcina barkeri, WP 011305188.1
Nautilia profundicola, WP 012663705.1
Lebetimonas sp. JH292, WP 024790055.1
Nitratifractor salsuginis, WP 013554774.1
Nitratiruptor sp. SB1552, WP 012082092.1
Sulfurovum riftiae, WP 067331915.1
Sulfurimonas autotrophica OK10, WP 013327834.1
Sulfurimonas paralvinellae GO25, QOP45928
Sulfurovum lithotrophicum, WP 046551692.1
Sulfurovum sp. NBC371, WP 011979956.1
Rhodospirillum rubrum, WP 011389179.1
D9 bin7, 1823
Thiogranum sp. MCG6865004.1
D10 bin17, 2938
Thiodiazotropha endolucinida, WP 069126330.1
Nitrospirota bacterium, MBI5142371
D4 bin8, 1062
Chromatiales bacterium, MCP52984461
Thiocapsa imhoffii, WP 200387263.1
D6 bin23, 1491
D10 bin17 3355
Thiococcus pfennigii, WP 200382210.1
D4 bin11, 299
Mariprofundus ferrooxydans, WP 009850258.1
Ghiorsea bivora, WP 038248777.1
D9 bin7, 1134
Gammaproteobacteria bacterium, MCB17242591
Sulfurivermis fontis, WP 127476803.1
Thiogranum longum, WP 132972667.1
D5 bin11, 2645
Burkholderiaceae bacterium, MBS11695731
D7 bin30, 795
Glaciimonas soli, WP 153232782.1
Noviherbaspirillum aerium, WP 151634103.1
Janthinobacterium sp. 17J80-10, WP 128902128.1
Group IV
Nitrospiraceae bacterium, RPI35857.1
D4 bin8, 2422
D4 bin11, 936
Nitrospira sp. MBH0177632.1
Candidatus Nitrospira nitrosa, WP 090743963.1
Lamellibrachia satsuma, QYZ66736.1
Lamellibrachia barhami, WP 304956550.1
D10 bin17, 4036
ex. Lucinisca nassula
Thiodiazotropha endolucinida, WP 069125479.1
D6 bin23, 1908
Massilia niastensis, WP 020653150.1
Acidithiobacillus ferridurans, WP 215879239.1
Noviherbaspirillum autotrophicum, WP 040040211.1
D7 bin30, 1199
Azovibrio restrictus, WP 303784568.1
Betaproteobacteria bacterium MBK9116013.1
Thiogranum sp. MCG6865004.1
D9 bin7, 984
Sulfurimonas hongkongensis AST-10, WP 021286533.1
Nautilia profundicola, WP 015902315.1
Sulfurimonas indica NW8N, WP 188109518.1
D10 bin17, 611
Monitilora ramsayi, MCU7854006.1
D4 bin8, 95
D10 bin17, 2563
Lucinisca nassula, MBW9265514.1
Candidatus Thiodiazotropha endolucinida, WP 069121921.1
D10 bin17, 4360
Clathrolucina costata, MBT3045810.1
Ctena orbiculat, PVV11169.1
Candidatus Thiodiazotropha endolucinida, WP 069120690.1
Candidatus Thiodiazotropha endoloripes, WP 069005930.1
Myrtea spinifera, MCU7796420.1
D10 bin17, 511
D6 bin23, 49
Chromatiaceae bacterium, MCW55858351
Gammaproteobacteria bacterium, MCW8828781.1
Sulfurovum indicum, WP 197548534.1
Sulfurimonas sediminis S2-6, QOP43073
Sulfurimonas lithotrophica GYSZ_1, QFR48966
Nitratifractor salsuginis DSM 16511, BAE44405.1
Sulfuricurvum sp. MLSB, KFN391311
Sulfurimonas gotlandica GD1, WP 008339563.1
Arcobacter nitrofigilis, WP 013135164.1
Arcobacter butzleri, WP 012013089.1
Sulfurovum indicum, WP 197549814.1
Sulfurovum riftiae, WP 082791987.1
Sulfurovum sp. NBC371, WP 012083422.1
Sulfurimonas indica NW8N, WP 152183246.1
D10 bin20, 1159
Sulfurimonas sediminis S2-6, QOP43361
Sulfurimonas hydrogeniphila NW10, WP 151900574.1
Wolinella succinogenes, CAA463031
Sulfurovum sp. AR, WP 008242794.1
Sulfurimonas aquatica H1576, 1493
D8 bin28, 16
Sulfurovum indicum, WP 197547823.1
Sulfurimonas gotlandica GD1, WP 008339404.1
Hydrogenimonas thermophila, BAH020181
Sulfurimonas denitrificans DSM1251, YP 393947
Sulfurimonas hongkongensis AST-10, WP 021286544.1
Sulfurimonas xiamenensis 1-1N, QFR43606
D8 bin 38, 284
Sulfurovum sp. AR, WP 008243064.1
D10 bin20, 2016
D8 bin28, 163
Sulfuricurvum sp. MLSB, WP 037949252.1
Sulfuricurvum kujiense, WP 013460913.1
Sulfurovum riftiae, WP 067328356.1
Sulfurovum indicum, WP 197547811.1
Sulfurovum sp. NBC37-1, WP 012083413.1
Sulfurimonas marina B2, QOP41360
Arcobacter nitrofigilis, WP 01313385.1
Sulfurimonas aquatica H1576, 1505
Sulfurimonas autotrophica OK10, WP 013327110.1
Sulfurimonas xiamenensis 1-1N, QFR43468
Sulfurimonas lithotrophica GYSZ_1, QFR48381
Sulfurimonas gotlandica GD1, WP 008339553.1
Group I
Tree scale: 0.1

Fig. S17. Maximum-likelihood tree of amino acid sequences of [NiFe]-hydrogenase large subunit, which are markers for hydrogen oxidation. The tree shows sequences from mangrove sediments metagenome-assembled genomes obtained in this study (different colors for group I, II, and IV) alongside representative reference sequences (black). Only bootstrap values ≥ 50% (based on 1000 bootstrap replicates) are shown at nodes as closed circles.

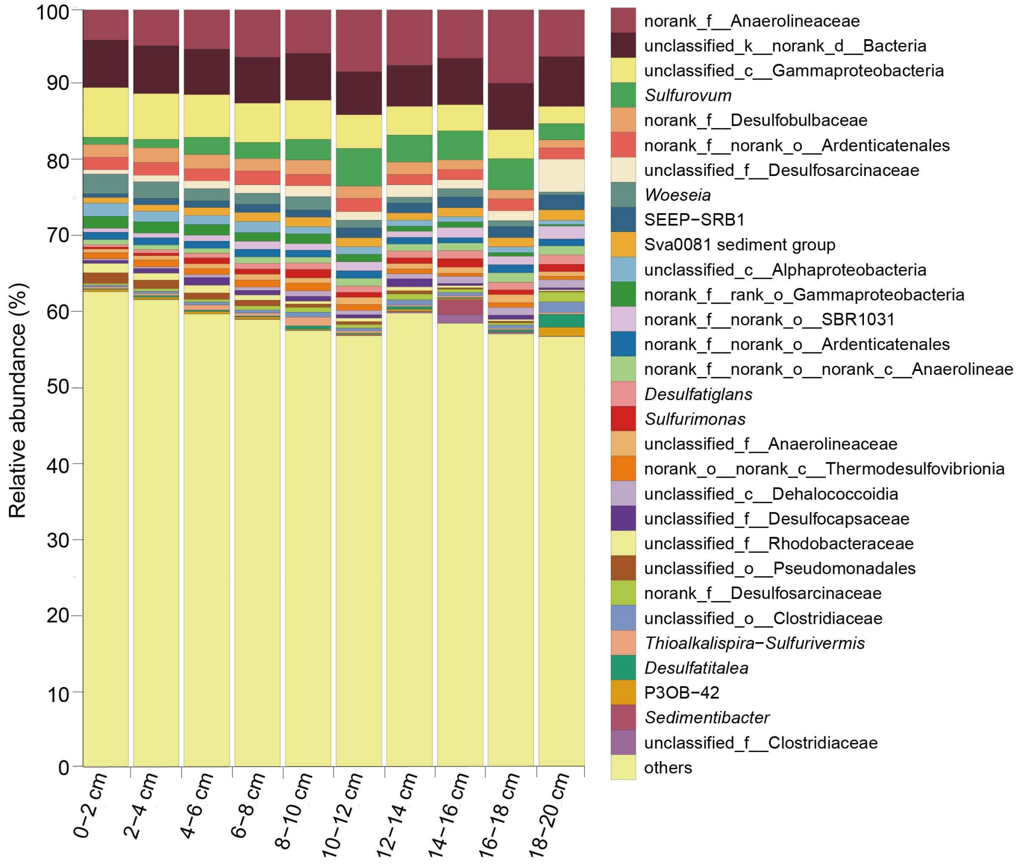

Fig. S18. Taxonomic compositions of microbial communities at the genus level based on 16S rRNA gene fragments extracted from metagenomes using the phyloFlash pipeline. For each plot, all taxa present in a relative abundance of ≥ 1.0% in at least one of the samples are shown, whereas those present in < 1.0% relative abundance are grouped in the "others" category.

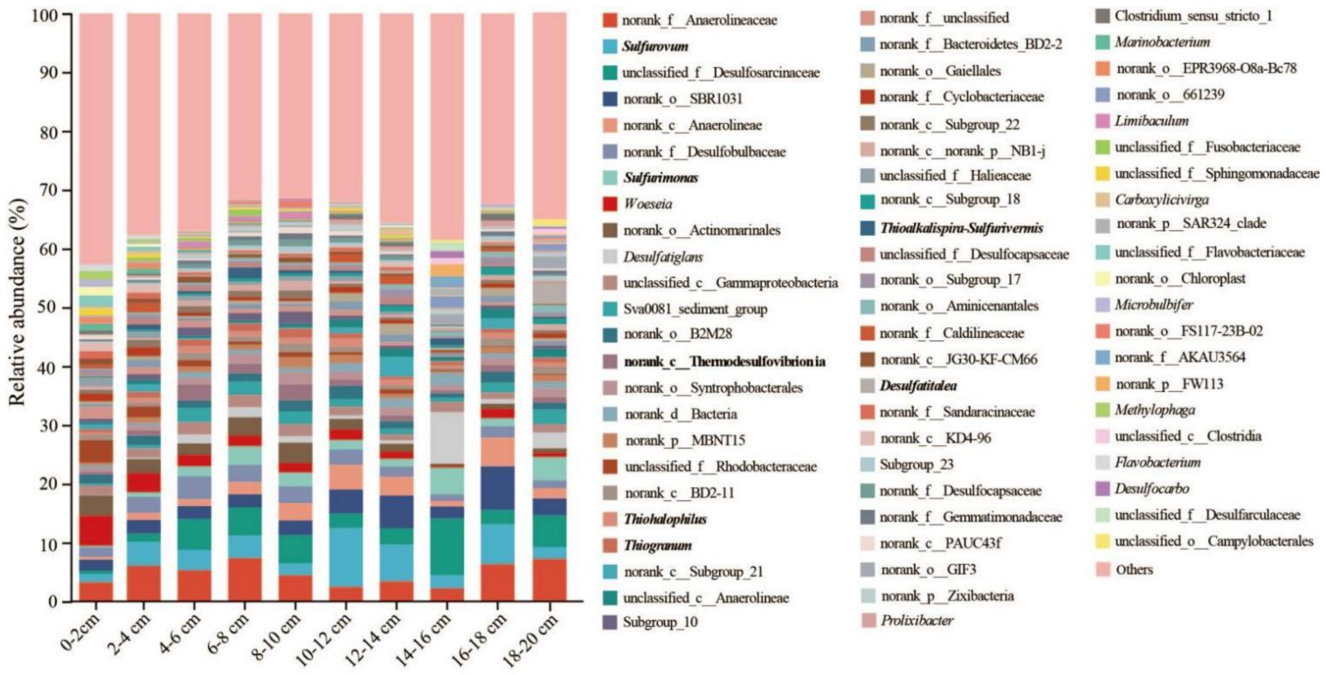

Fig. S19. Taxonomic distribution produced by 16S rRNA gene amplicon sequencing among the ten mangrove sediments at the genus level. For each plot, all taxa present in a relative abundance of ≥ 1.0% in at least one of the samples are shown, whereas those present in < 1.0% relative abundance are grouped with unassigned sequences in the “others” category. The bold fonts represent the chemolithoautotrophic taxa.

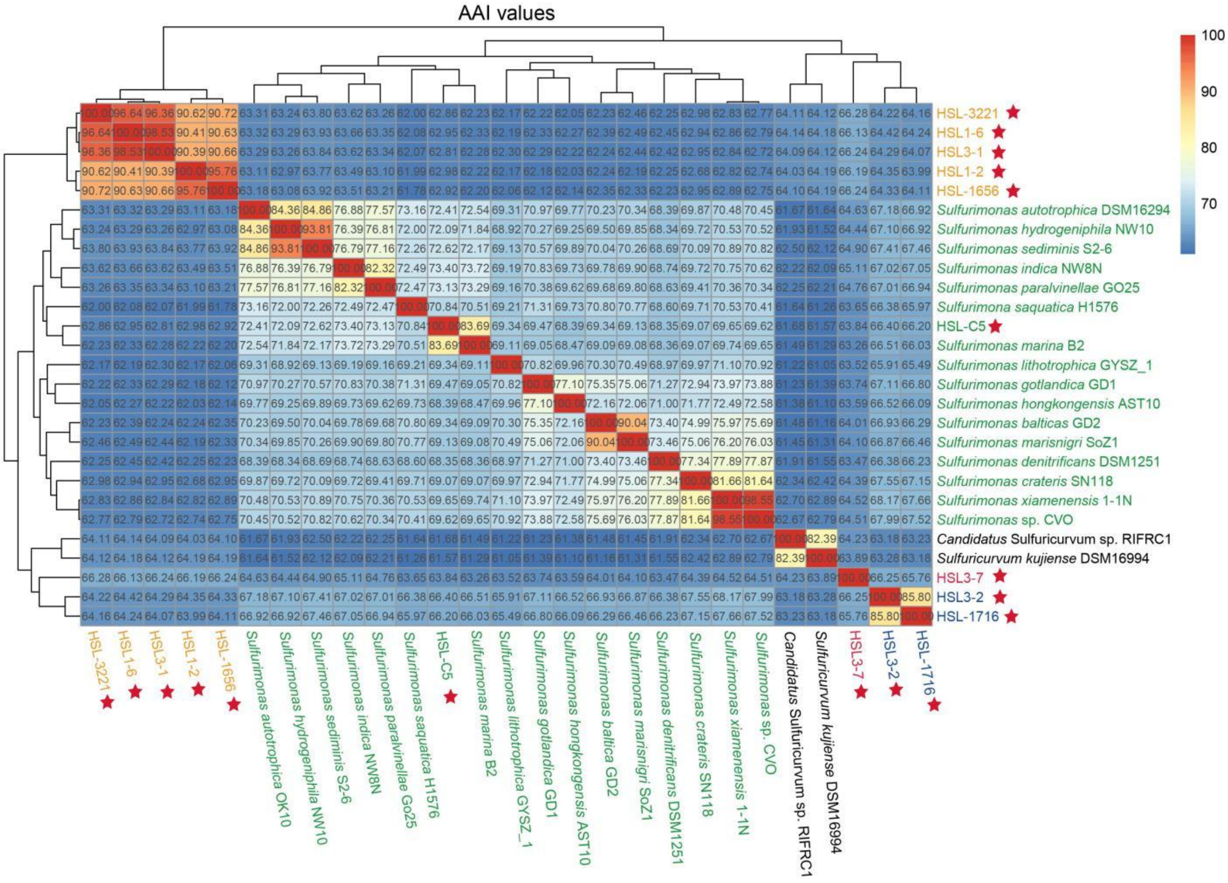

Fig. S20. AAI values between genomes of ten isolates and genomes of type strains of *Sulfurimonas* in the phylum *Campylobacterota*. The asterisk represents the strains isolated in this study.

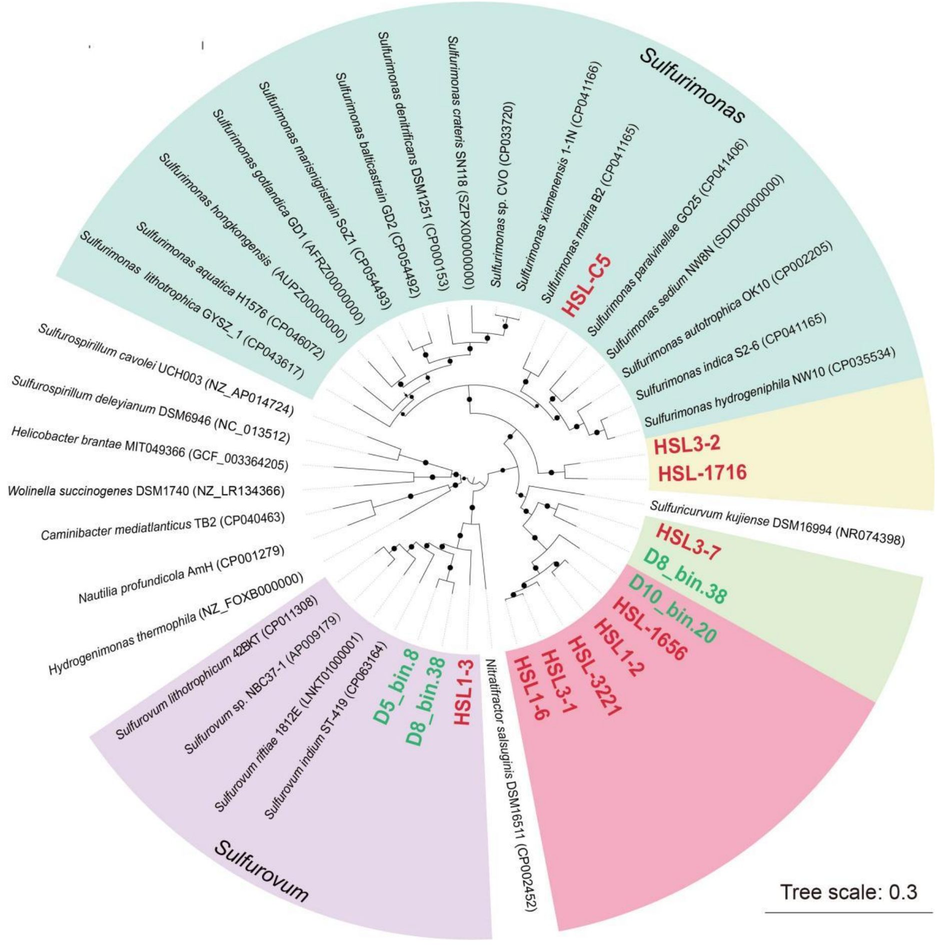

Fig. S21. The maximum-likelihood phylogenetic tree of ten pure cultures from *Campylobacterota* and their closely related taxa, based on the whole-genome sequence data. The red font indicates the isolates in this study, and the green font indicates the *Campylobacterota* MAGs from this study. The background colors of red, yellow, and light green represent three putative new genera. The bar represents 0.3 substitutions per position.

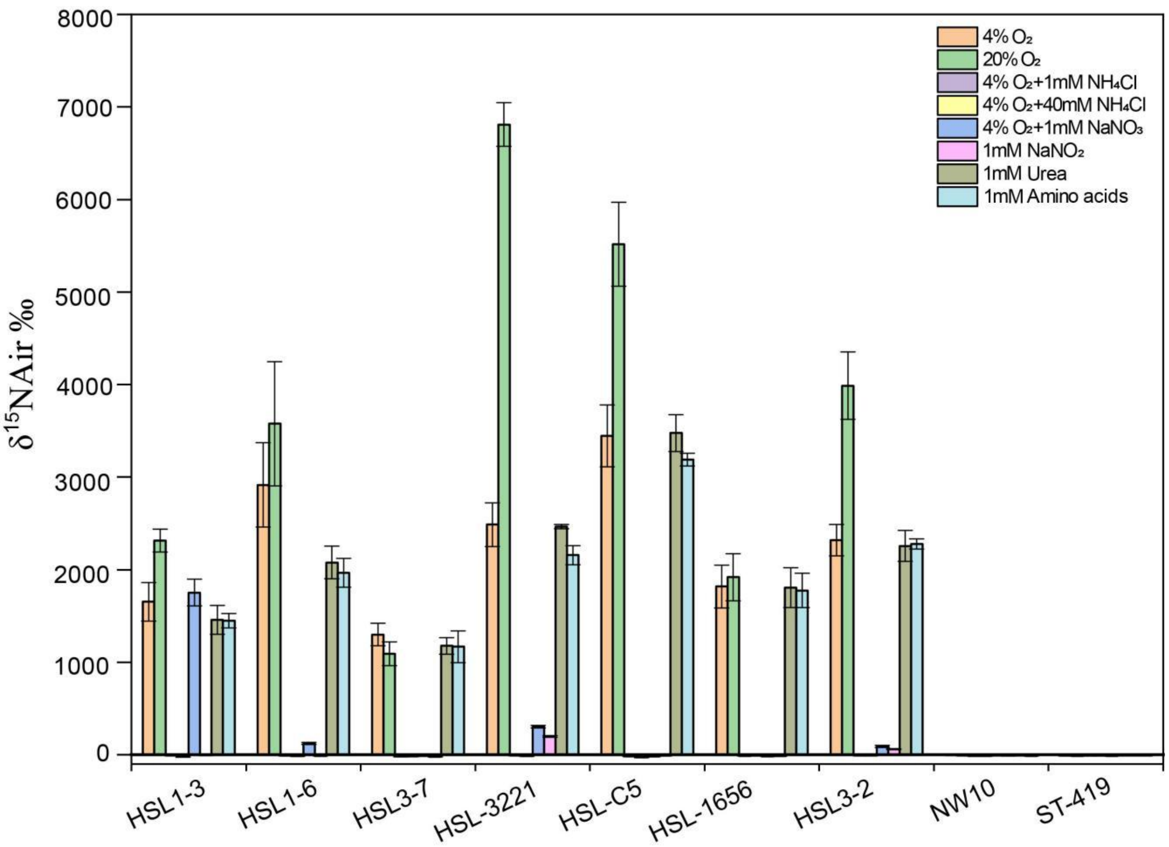

Fig. S22. Nitrogen fixation activities from representative strains in the presence of different oxygen concentration and different inorganic or organic nitrogenous compounds. Standard deviations are indicated by error bars.
